# Cancer‐Associated Fibroblasts Promote Tumor Immunosuppression in Hepatocellular Carcinoma via the NNMT‐ANGPTL4 Axis

**DOI:** 10.1002/advs.202521418

**Published:** 2026-04-14

**Authors:** Shounan Lu, Shanjia Ke, Hongjun Yu, Zhanzhi Meng, Miaoyu Bai, Yanan Xu, Hui Zhu, Jinwen Yang, Baolin Qian, Bing Yin, Chaoqun Wang, Zhigang Feng, Zhongyu Li, Yongzhi Zhou, Zihao Li, Xinglong Li, Yongliang Hua, Yao Fu, Wei Tang, Yaohua Wu, Yong Ma

**Affiliations:** ^1^ Department of Minimally Invasive Hepatic Surgery The First Affiliated Hospital of Harbin Medical University Harbin China; ^2^ Key Laboratory of Hepatosplenic Surgery Ministry of Education The First Affiliated Hospital of Harbin Medical University Harbin China; ^3^ Department of Hepatobiliary Surgery Peking University People's Hospital Beijing China; ^4^ Department of Anesthesiology Taizhou First People’s Hospital Taizhou China; ^5^ Department of Hepatopancreatobiliary Surgery Affiliated Hangzhou First People's Hospital Zhejiang University School of Medicine Hangzhou China; ^6^ Department of Hepatobiliary Surgery The Second Affiliated Hospital of Army Medical University Chongqing China; ^7^ The First Department of General Surgery Affiliated Hospital of Inner Mongolia Minzu University Tongliao China; ^8^ Department of Pediatric Surgery The First Affiliated Hospital of Harbin Medical University Harbin China; ^9^ Department of Ultrasound The First Affiliated Hospital of Harbin Medical University Harbin China; ^10^ International Health Care Center National Center for Global Health and Medicine Tokyo Japan; ^11^ Hepato‐Biliary‐Pancreatic Surgery Division Department of Surgery The University of Tokyo Hospital Tokyo Japan; ^12^ Department of Thyroid Surgery The First Affiliated Hospital of Harbin Medical University Harbin China

**Keywords:** ANGPTL4, HCC, histone lactylation, Immune evasion, NNMT

## Abstract

**Background & Aims**: Cancer‐associated fibroblasts (CAFs) drive immunosuppression in hepatocellular carcinoma (HCC). However, their metabolic regulation remains poorly defined. We investigated the role of nicotinamide N‐methyltransferase (NNMT) in CAFs. **Approach & Results**: High NNMT expression in CAF tissues was confirmed by western blotting and immunofluorescence staining. Primary CAFs from HCC patients, single‐cell RNA‐seq (GSE149614), patient‐derived organoids (PDOs), and fibroblast‐specific NNMT‐knockout mice were integrated by metabolomic analyses. NNMT in CAFs binds EZH2 and impedes its nuclear translocation, thereby reducing H3K27me3 enrichment at the promoter of angiopoietin‐like 4 (ANGPTL4) to increase ANGPTL4 secretion. Secreted ANGPTL4 engages GLUT1 in HCC cells, activating aerobic glycolysis and increasing histone H3K18la levels. This epigenetic reprogramming transcriptionally upregulates PD‐L1 expression, thereby facilitating tumor immune evasion. Additionally, CAF‐derived ANGPTL4 promotes angiogenesis in HCC. Therapeutically, targeting the NNMT‐ANGPTL4 axis restored CD8^+^ T‐cell activity and synergized with anti‐PD‐L1 therapy in both patient‐derived xenografts (PDXs) and fibroblast‐specific NNMT‐knockout murine models. **Conclusion**: We identified an NNMT‐ANGPTL4‐driven metabolic‐epigenetic cascade in CAFs that induces PD‐L1‐mediated immune evasion, providing a therapeutic strategy to overcome resistance to immunotherapy in patients with HCC.

## Graphical abstract

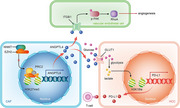

**Abbreviations**
ANGPTL4: angiopoietin‐like 4
CAFs: Cancer‐associated fibroblasts
CM: conditioned media
ECM: extracellular matrix
HCC: Hepatocellular carcinoma
NFs: normal fibroblasts
NNMT: Nicotinamide N‐methyltransferase
PD‐1: programmed cell death protein 1
PD‐L1: programmed cell death‐ligand 1
PDO: patient‐derived organoid
PDX: patient‐derived xenograft
TME: tumor microenvironment

## 1 Introduction

Hepatocellular carcinoma (HCC) is a prevalent malignancy, with 70 %–80 % of patients presenting at advanced stages upon diagnosis. While combined local and systemic therapies aim to extend survival in advanced HCC, the efficacy of targeted agents remains suboptimal, and numerous novel drug trials have encountered clinical failure [1, 2]. Consequently, identifying novel therapeutic targets and developing more effective treatment strategies are critical for HCC management.

The tumor microenvironment (TME) constitutes a multicellular ecosystem characterized by intricate tumor‐stroma crosstalk, wherein cancer‐associated fibroblasts (CAFs) serve as pivotal regulatory components [3]. CAFs drive HCC progression through direct modulation of cancer cells via soluble factors and exosomes, as well as indirect facilitation via extracellular matrix (ECM) remodeling. Furthermore, CAFs enhance malignant phenotypes while recruiting immunosuppressive cells such as neutrophils and monocytes, thereby establishing an immune‐tolerant niche that promotes tumor immune evasion [4, 5]. Nicotinamide N‐methyltransferase (NNMT), a member of the N‐methyltransferase family, has emerged as a significant focus in oncological research owing to its tumorigenic role in regulating nicotinamide methylation [6]. Studies have consistently demonstrated that NNMT is upregulated across diverse malignancies, including colorectal, gastric, and ovarian cancers, indicating that NNMT is a prominent tumor biomarker [7, 8, 9]. Previous investigations have identified NNMT as a core metabolic regulator in CAFs [9], whereas our prior work further elucidated its function in driving intrahepatic cholangiocarcinoma progression. Despite the established hepatic abundance of NNMT, its specific biological functions in HCC‐associated CAFs remain poorly characterized and merit rigorous investigation.

In this study, we revealed that NNMT is highly expressed in CAFs and mediates the immune evasion of HCC cells. Further investigation revealed that NNMT can promote the secretion of ANGPTL4, a cytokine that structurally resembles angiogenin and plays important roles in glucose metabolism, lipid metabolism, the inflammatory response, and angiogenesis. ANGPTL4 derived from CAFs was shown to activate aerobic glycolysis and promote PD‐L1 expression in HCC cells, thereby mediating immune evasion. In addition, ANGPTL4 was shown to promote angiogenesis and synergistically contribute to tumor progression. Our findings elucidate the complex role of the NNMT‐ANGPTL4 axis in promoting immunosuppression and angiogenesis in CAFs in HCC, establishing the NNMT–ANGPTL4 axis as a critical therapeutic target for increasing immunotherapeutic efficacy.

## 2 Results

### 2.1 NNMT is Highly Expressed in CAFs and Associated With Poor Prognosis in Patients With HCC

**FIGURE 1:**
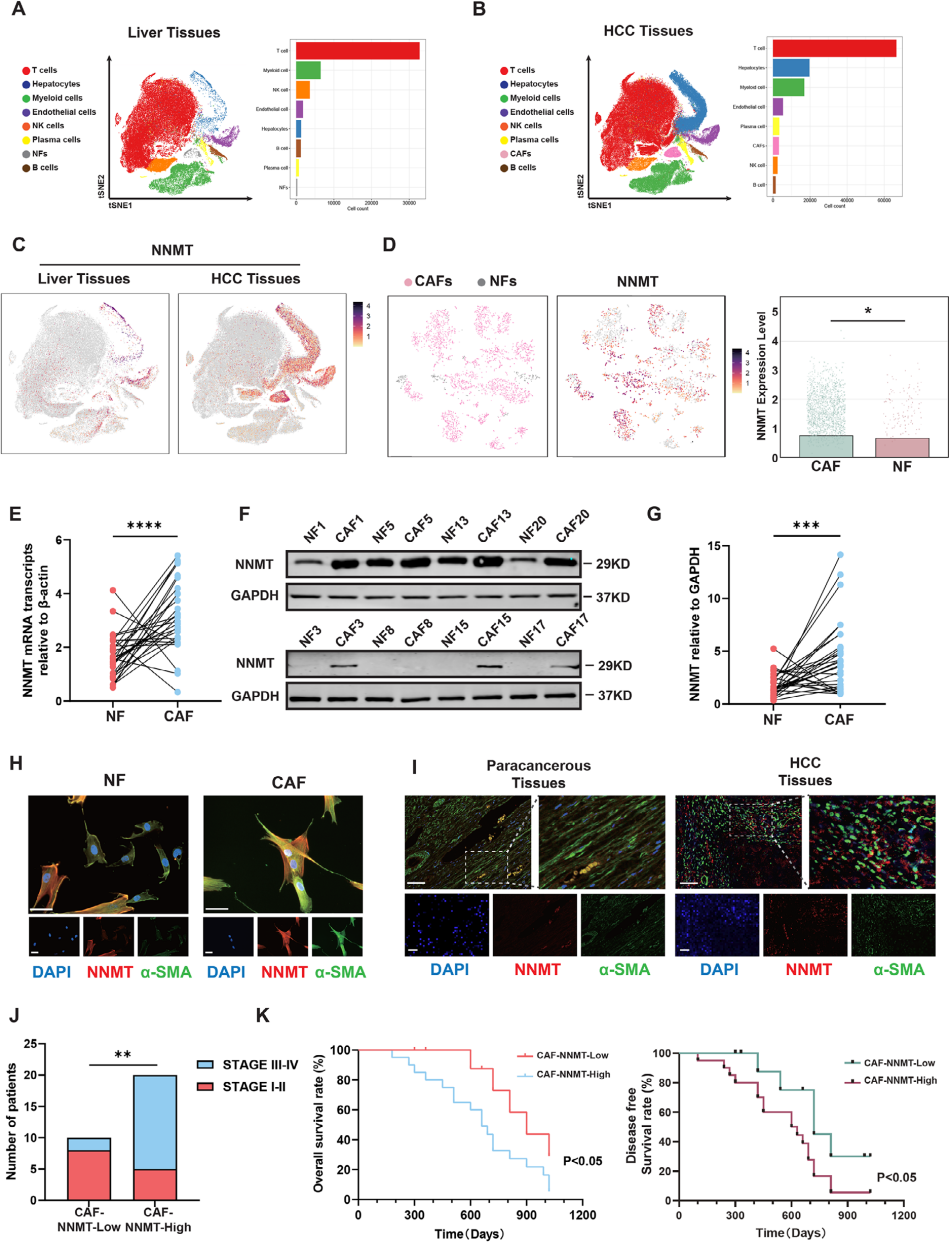
NNMT is expressed at high levels in CAFs. (A) TSNE plot of 8 main cell types from normal liver tissues in the GSE149614, GSE146115, GSE151530, and GSE189903 datasets. (B) TSNE plot of 8 main cell types from HCC tissues in the GSE149614, GSE146115, GSE151530, and GSE189903 datasets. (C) TSNE plot color‐coded to display NNMT expression levels (ranging from gray to red) across different cell types. (D) Differential expression of NNMT in the GSE149614, GSE146115, GSE151530 and GSE189903 datasets, showing expression in NFs and CAFs; (E–G) The expression levels of NNMT mRNA (E) and protein (F) in NFs and CAFs (n = 30); (H) Representative images of NNMT immunofluorescence staining in NFs and CAFs; (I) Representative images of NNMT and α‐SMA staining in HCC and adjacent tissues; (J) Percentage of AJCC stage I–IV HCC patients with low or high NNMT expression in CAFs (n = 30); (K) OS and DFS for patients based on low/negative or high NNMT expression in CAFs (n = 30). (^*^ *p*<0.05, ^**^ *p* < 0.01, ^***^ *p* < 0.001, ^****^ *p* < 0.0001).

Analysis of GEO single‐cell sequencing data (GSE149614, GSE146115, GSE151530, GSE189903) revealed differential enrichment of NNMT expression in CAFs vs. normal fibroblasts (NFs) (Figure 1A–D). Primary CAFs and NFs isolated from HCC patients presented significantly elevated NNMT protein and mRNA levels in CAFs relative to those in NFs (Figure 1E–G; Figure S1A), with immunofluorescence staining confirming intensified NNMT signals in CAFs (Figure 1H). Tissue analysis also revealed heightened NNMT expression in HCC‐derived CAFs compared with that in fibroblasts from adjacent normal tissues (Figure 1I). Subsequent stratification of the patients with HCC by their CAF‐NNMT expression levels revealed that high NNMT expression correlated significantly with advanced TNM stage and reduced survival (Figure 1J,K).

### 2.2 High Expression of NNMT in CAFs can Promote the Proliferation and Immune Escape of HCC Cells

**FIGURE 2:**
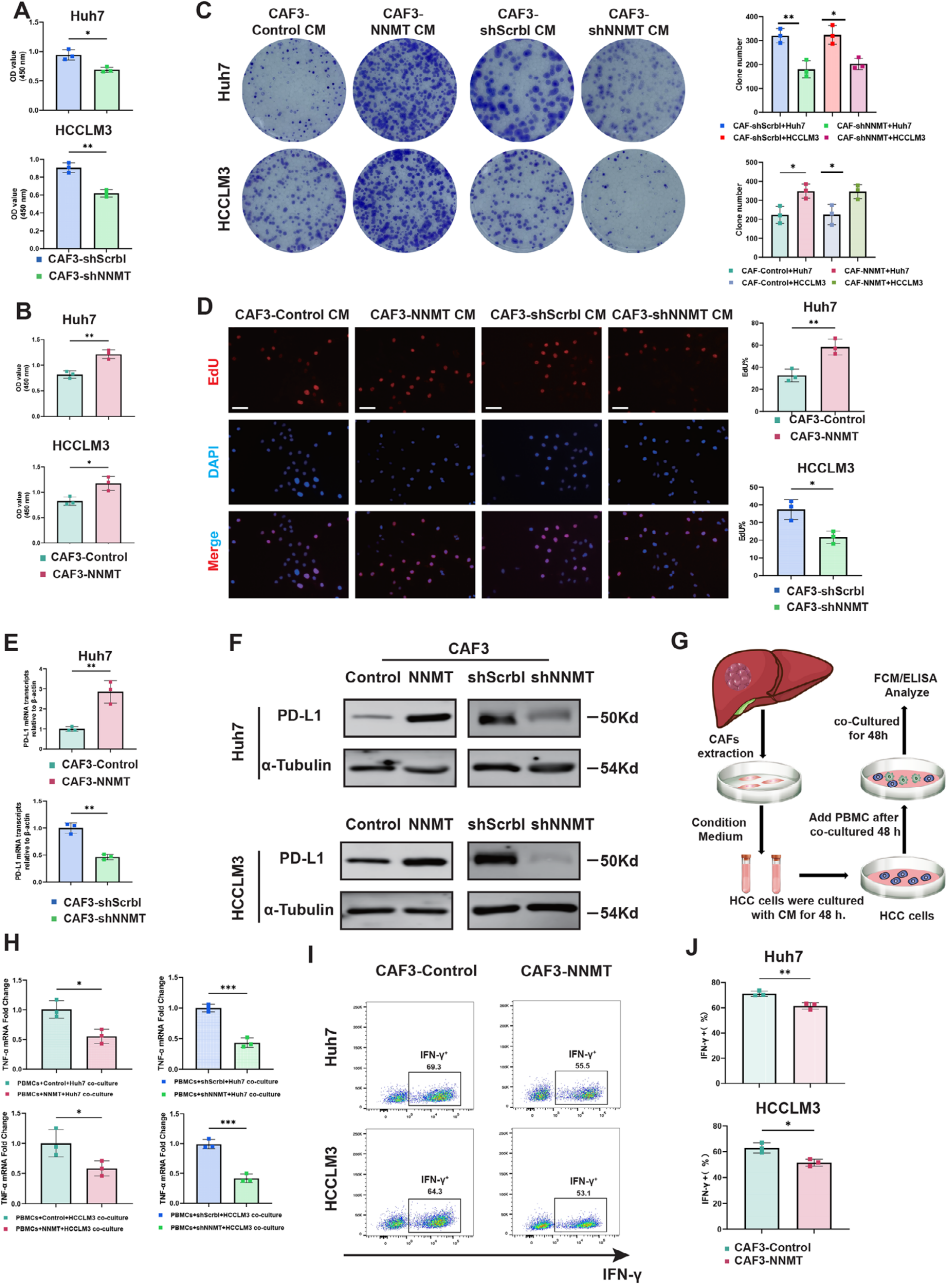
High levels of NNMT in CAFs promote HCC proliferation and PD‐L1 expression. (A,B) CCK‐8 assays were used to evaluate the proliferation of Huh7 and HCCLM3 cells after treatment with shNNMT‐CAF (A) or NNMT‐CAF (B) supernatant. (C) Representative images of colony formation assays for Huh7 and HCCLM3 cells treated with NNMT‐CAF or shNNMT‐CAF supernatant. (D) EdU proliferation assay images of Huh7 and HCCLM3 cells after treatment. (E) qPCR analysis of PD‐L1 expression in Huh7 cells post‐treatment. (F) Western blot analysis of PD‐L1 expression in Huh7 and HCCLM3 cells following treatment with the CAF supernatant. (G) Effect of different conditions on T‐cell activity in PBMCs. (H) TNF‐α mRNA levels and I‐J IFN‐γ levels in CD8^+^ T cells. (n = 3; data are presented as the mean ± SD; ^*^ *p* < 0.05, ^**^ *p* < 0.01, ^***^ *p* < 0.001.).

Lentiviral vectors for NNMT overexpression and knockdown were constructed and transfected into primary CAFs (Figure S1B,C). Conditioned media (CM) from these modified CAFs were applied to Huh7 and HCCLM3 HCC cell lines. Functional assays demonstrated that CM from NNMT‐overexpressing CAFs promoted HCC cell proliferation (CCK‐8 and colony formation assays: Figure 2A–C), whereas CM from NNMT‐knockdown CAFs inhibited proliferation, as further confirmed by EdU assays (Figure 2D; Figure S2A).

Given the established role of CAFs in promoting tumor immune evasion, particularly through PD‐L1/PD‐1 axis‐mediated T‐cell exhaustion [10, 11], we examined PD‐L1 expression in CM‐treated HCC cells. NNMT overexpression in CAFs significantly upregulated PD‐L1 mRNA and protein expression levels in recipient HCC cells; conversely, NNMT knockdown suppressed PD‐L1 expression (Figure 2E,F; Figure S2B,C). To assess the immunomodulatory effects, CM‐treated HCC cells were co‐cultured with peripheral blood mononuclear cells (PBMCs) for 48 h, with TNF‐α expression serving as a CD8^+^ T‐cell activity indicator (Figure 2G). These HCC cells significantly suppressed TNF‐α expression in PBMCs (Figure 2H). Flow cytometric analysis further revealed reduced frequency of IFN‐γ‐producing CD8^+^ T cells (CD8^+^IFN‐γ+) among PBMCs following co‐culture with CM‐treated HCC cells (Figure 2I,J). Collectively, these results reveal that NNMT overexpression in CAFs increases PD‐L1 expression in HCC cells and facilitates tumor immune evasion.

### 2.3 NNMT Promotes ANGPTL4 Secretion by CAFs

**FIGURE 3:**
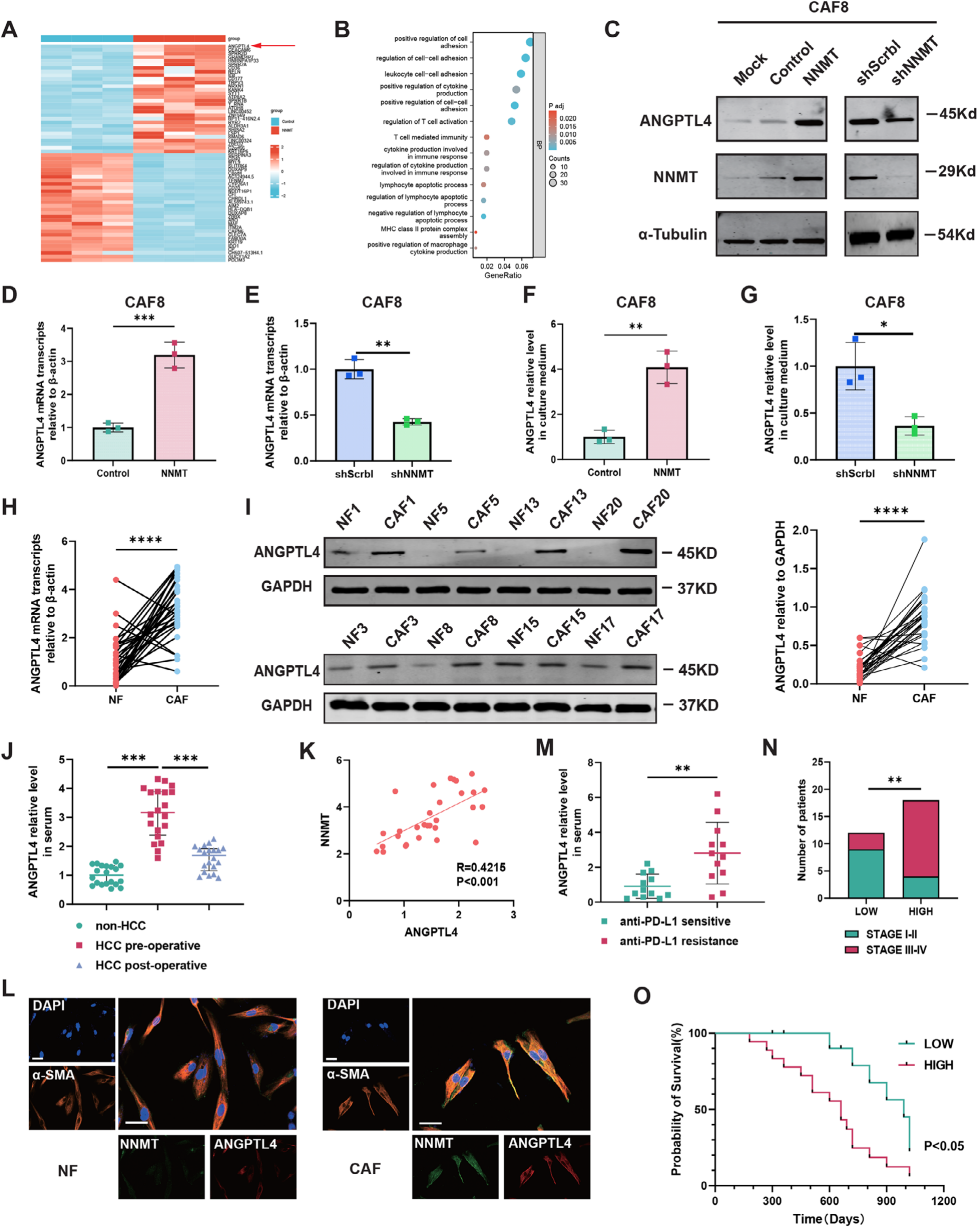
NNMT promotes ANGPTL4 secretion in CAFs. (A) Transcriptomic sequencing of CAFs transfected with NNMT‐overexpressing and control lentiviral vectors. (B) KEGG pathway analysis of the transcriptome data. (C) Western blot analysis of ANGPTL4 protein levels in NNMT‐transfected CAFs. (D,E) ANGPTL4 mRNA levels in CAFs after NNMT overexpression (D) or knockdown (E). (F,G) ANGPTL4 levels in the culture medium of transfected CAFs. (H) ANGPTL4 mRNA levels in CAFs and NFs (n = 30). (I) Representative Western blot images of ANGPTL4 in CAFs and NFs. (J) ANGPTL4 levels in the serum of healthy individuals and patients with HCC. (K) Correlations between NNMT and ANGPTL4 expression. (L) Representative immunofluorescence images of NNMT and ANGPTL4 in CAFs. (M) Serum ANGPTL4 levels in anti‐PD‐L1‐sensitive and anti‐PD‐L1‐resistant patients. N AJCC stage I‐IV HCC patients stratified by ANGPTL4 expression. O Kaplan–Meier curves stratified by ANGPTL4 expression. (n = 3; data are presented as the mean ± SD; ^*^ *p* < 0.05, ^**^ *p* < 0.01, ^***^ *p* <0.001.).

Transcriptomic sequencing of NNMT‐overexpressing vs. control CAFs was performed to elucidate the role of NNMT in CAF‐mediated HCC progression and immune evasion. This analysis revealed angiopoietin‐like 4 (ANGPTL4) as the most significantly upregulated transcript in NNMT‐modified CAFs, with GO enrichment confirming its association with tumor immunity (Figure 3A,B). We hypothesized that NNMT drives HCC progression through increased ANGPTL4 secretion. Validation experiments revealed that NNMT overexpression substantially increased ANGPTL4 mRNA/protein expression and secretory output, whereas NNMT knockdown abrogated these effects (Figure 3C–G). Notably, compared with normal fibroblasts (NFs), primary CAFs exhibited intrinsically elevated ANGPTL4 expression and secretion (Figure 3H,I). Peripheral blood analysis revealed that compared with healthy individuals, patients with HCC had significantly elevated ANGPTL4 levels, which markedly decreased one‐week post‐resection (Figure 3J). In CAFs, NNMT expression positively correlated with ANGPTL4 expression (Figure 3K), and immunofluorescence staining confirmed their co‐localization (Figure 3L). When patients were stratified by anti‐PD‐L1 therapeutic response, patients with resistance cases exhibited higher serum ANGPTL4 levels than their sensitive counterparts did (Figure 3M). Prognostic analysis revealed that patients with high ANGPTL4 expression showed an advanced TNM stage and reduced survival (Figure 3N,O).

### 2.4 NNMT Inhibits the Nuclear Translocation of EZH2 and Suppresses H3K27me3 Levels on the Promoter Region of ANGPTL4

**FIGURE 4:**
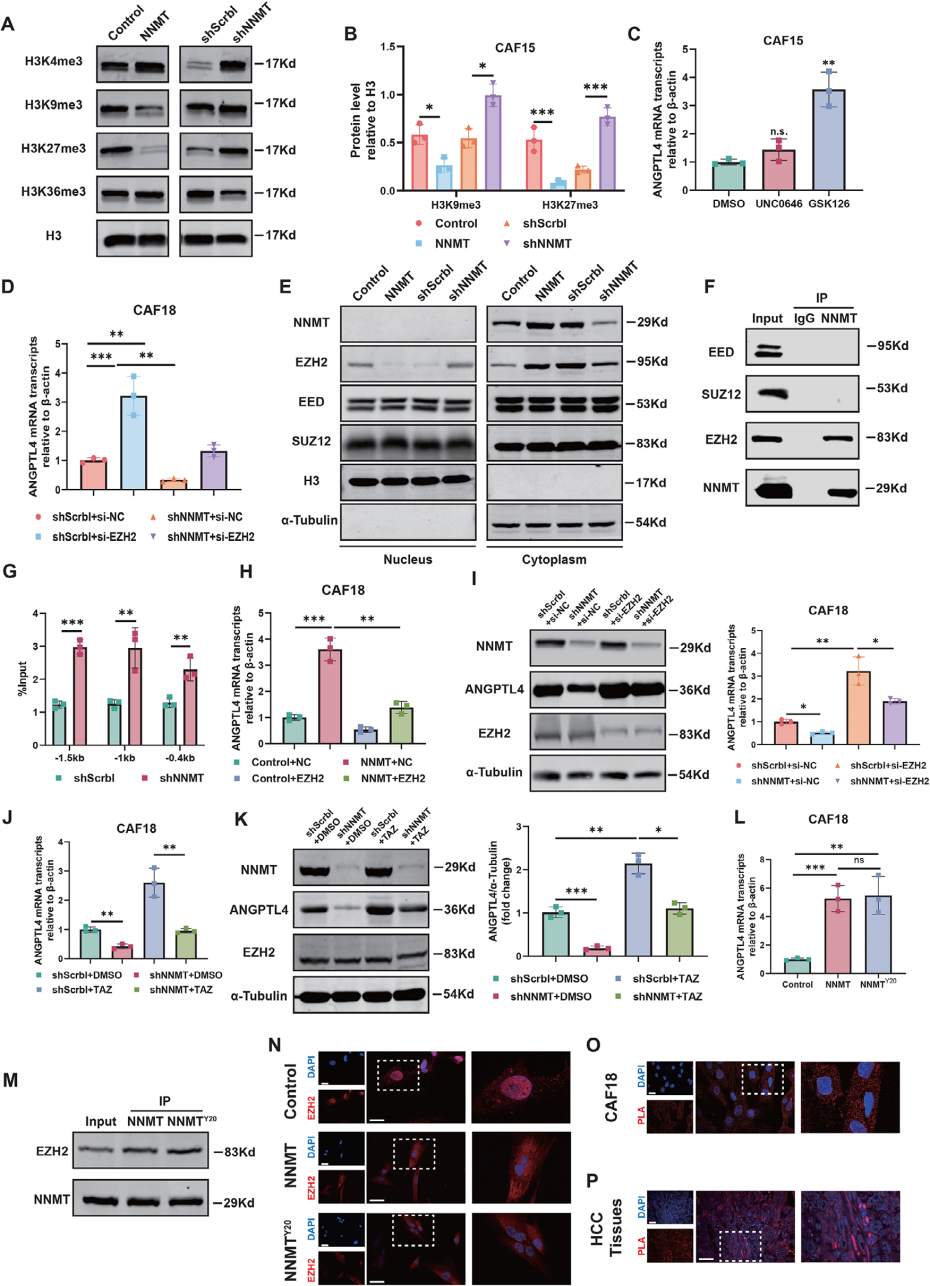
NNMT binding to EZH2 promotes the expression of ANGPTL4 in CAFs. (A,B) WB analysis of the effect of NNMT on histone modifications. (C) qPCR of ANGPTL4 mRNA in CAFs treated with 5 µM UNC0646 or 5 µM GSK126. (D) qPCR analysis of ANGPTL4 mRNA in CAFs transfected with shNNMT and/or si‐EZH2. (E) Western blot analysis of NNMT, EZH2, EED, and SUZ12 levels in the nucleus and cytoplasm of CAFs. (F) Co‐IP analysis of NNMT binding to the PRC2 complex in CAFs. (G) ChIP analysis of H3K27me3 enrichment at the ANGPTL4 promoter. (H) Effects of EZH2 and NNMT on ANGPTL4 mRNA levels. (I) Effects of si‐EZH2 and/or shNNMT on ANGPTL4 protein (left) and mRNA (right) levels. J–K Effects of TAZ and shNNMT on ANGPTL4 mRNA (J) and protein (K) expression. (L) Effects of the NNMTY20 mutation on ANGPTL4 mRNA levels. (M) Effect of the Y20 mutation on the ability of NNMT to bind to EZH2. (N) Representative images of EZH2 immunofluorescence staining in CAFs. (O,P) PLA probe was used to detect NNMT and EZH2 binding in CAFs (O) and HCC tissues (P). (n = 3; data are presented as the mean ± SD; ^*^ *p* < 0.05, ^**^ *p* < 0.01, ^***^ *p*<0.001.).

While previous studies have shown that NNMT depletes S‐adenosylmethionine (SAM) to inhibit global histone methylation [12], we postulated that NNMT plays a specific role in regulating ANGPTL4 transcription via promoter histone modifications. Screening of key histone marks in CAFs revealed that NNMT knockdown increased H3K9me3 and H3K27me3 levels, whereas NNMT overexpression suppressed these repressive marks, with the levels of other methylation sites remaining unaltered (Figure 4A,B; Figure S3A). Subsequent application of selective methylation inhibitors revealed that H3K27me3 blockade (GSK126) substantially increased ANGPTL4 mRNA levels, whereas H3K9me3 inhibition (UNC0646) elicited moderate effects (Figure 4C).

Although NNMT knockdown marginally reduced SAM levels in CAFs (statistically non‐significant), exogenous SAM supplementation failed to reverse NNMT‐overexpression‐induced upregulation of ANGPTL4 expression (Figure S3B,C), suggesting that SAM‐dependent mechanisms in ANGPTL4 transcriptional activation are not involved. Given the catalytic role of the PRC2 complex in H3K27me3 deposition, we hypothesized that NNMT modulates this histone mark through PRC2 regulation. Validation assays confirmed the unchanged expression of PRC2 core components (EED, SUZ12, and EZH2) following NNMT manipulation (Figure S3D). Next, we constructed si‐EZH2, si‐EED, and si‐SUZ12 and verified their efficiencies and their effects on H3K27me3 in cells (Figure S3G–I). Intriguingly, both NNMT knockdown and PRC2 subunit depletion (si‐EED/si‐SUZ12/si‐EZH2) increased ANGPTL4 expression (Figure 4D; Figure S3E,F).

Because PRC2 inhibition promoted ANGPTL4 expression, whereas NNMT did not alter PRC2 protein levels, we hypothesized that NNMT impairs PRC2 function via subcellular redistribution. NNMT overexpression shifted EZH2 from the nuclear compartment to the cytoplasmic compartment, whereas NNMT knockdown produced reciprocal effects, with EED/SUZ12 localization remaining unaffected (Figure 4E). Coimmunoprecipitation confirmed direct binding between NNMT and EZH2 (Figure 4F). Furthermore, the results of the IP assay revealed that overexpression of NNMT did not affect the binding of EZH2 to SUZ12 or EED (Figure S3J,K). We then mapped the domains of NNMT and EZH2 required for their interaction by expressing different truncation mutations of EZH2 and NNMT in HEK293T cells. The results indicated that the 58–199 aa domain of NNMT was required for its binding to EZH2 (Figure S4A) and that the C‐terminal domain (504‐751 aa) of EZH2 was necessary for its binding to NNMT (Figure S4B). These findings demonstrate that NNMT, by binding to EZH2, inhibits its nuclear entry but does not affect its binding to SUZ12 and EED.

Chromatin immunoprecipitation (ChIP) further demonstrated that NNMT knockdown increased EZH2 occupancy and H3K27me3 enrichment at the ANGPTL4 promoter (Figure 4G; Figure S5A), supporting NNMT‐mediated control of EZH2 nuclear trafficking. Rescue experiments confirmed that EZH2 overexpression abrogated NNMT‐driven upregulation of ANGPTL4 expression (Figure 4H,I; Figure S5B). Consistent with these results, the EZH2 inhibitor tazemetostat was shown to increase ANGPTL4 expression (Figure 4J,K). To decouple catalytic and scaffolding functions, we transfected CAFs with a catalytically dead NNMTY20 mutant (Figure S5C,D). This mutant retained its H3K27me3 suppression capacity and promoted ANGPTL4 transcription (Figure 4L; Figure S5E) while maintaining EZH2 binding (Figure 4M). To investigate whether the NNMTY20 mutation affects the overall conformation of NNMT, we examined the binding of the NNMTY20 mutant to its known interacting partner, PRDX6 [13]. The results revealed that the NNMTY20 mutant retained the ability to bind to PRDX6, suggesting that this mutation does not induce a global conformational change in NNMT. Immunofluorescence staining revealed that both wild‐type NNMT and NNMTY20 induced nuclear exclusion of EZH2 (Figure 4N). Proximity ligation assays (PLAs) further verified the in situ NNMT–EZH2 interaction between CAFs and HCC tissues (Figure 4O,P).

### 2.5 ANGPTL4 Promotes PD‐L1 Expression in HCC Cells and Increases Immune Evasion

**FIGURE 5:**
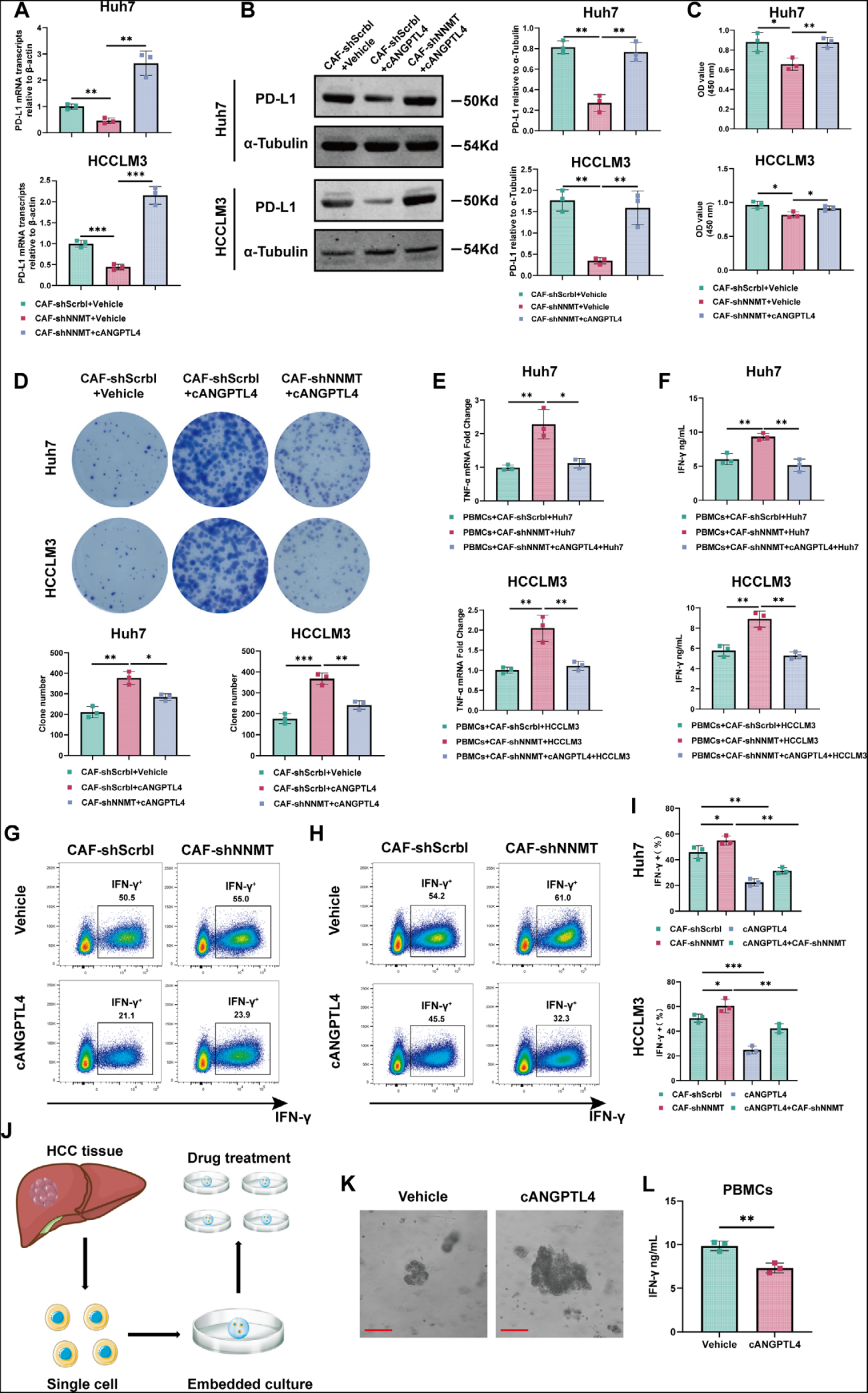
ANGPTL4 promotes PD‐L1 expression in HCC cells and increases immune evasion. (A) qPCR analysis of PD‐L1 expression in Huh7 (up) and HCCLM3 (down) cells after treatment with supernatant from NNMT‐knockdown CAFs and cANGPTL4. (B) Western blot analysis of PD‐L1 expression in Huh7 and HCCLM3 cells under the same conditions. (C) CCK‐8 assays were used to evaluate the proliferation of Huh7 and HCCLM3 cells after treatment. (D) Representative image of colony formation experiments for Huh7 and HCCLM3 cells. (E) TNF‐α mRNA levels in PBMCs treated under various conditions. (F–I) HCC cells treated with conditioned media (CM) from CAFs transfected with shNNMT or with cANGPTL4 were cocultured with PBMCs. IFN‐γ expression and secretion were detected by ELISA (F) or flow cytometry (G–I). (J) Diagram for constructing the human hepatocellular carcinoma (HCC) organoid model. (K) Image of patient‐derived organoids after cANGPTL4 treatment. (L) IFN‐γ production in organoids with or without cANGPTL4 treatment. (n = 3; data are presented as the mean ± SD; ^*^ *p* < 0.05, ^**^ *p* < 0.01, ^***^ *p*<0.001.).

To determine whether CAFs promote PD‐L1 expression via ANGPTL4 secretion, recombinant ANGPTL4 (cANGPTL4) was applied to HCC cells. cANGPTL4 significantly upregulated PD‐L1 mRNA and protein expression levels (Figure 5A,B). The results of functional assessments demonstrated an increase in ANGPTL4‐mediated proliferation through CCK‐8, colony formation (Figure 5C,D), and EdU (Figure S6A) assays. In PBMC‐HCC co‐cultures supplemented with cANGPTL4, the PBMCs exhibited suppressed TNF‐α mRNA expression (Figure 5E; Figure S6B) and reduced IFN‐γ secretion (Figure 5F), indicating impaired T‐cell functionality. Complementary flow cytometry confirmed the ANGPTL4‐mediated suppression of IFN‐γ production in CD8^+^ T cells across the experimental conditions (Figure 5G–I). By co‐culturing Huh7 cells transfected with si‐PD‐L1 with PBMCs, we found that cANGPTL4 failed to further increase the levels of IFN‐γ and TNF‐α, which were not significantly different from those in the control group (Figure S6C). These findings indicated that knockdown of PD‐L1 abrogated the effect of cANGPTL4 on HCC cells, suggesting that its function is dependent on PD‐L1 expression. Using fresh HCC surgical specimens, we established patient‐derived organoids (PDOs) as clinically relevant models (Figure 5J). When PDOs were co‐cultured with PBMCs and cANGPTL4, CD8^+^ T‐cells exhibited significantly diminished IFN‐γ activity, indicating that the immunosuppressive capacity of these cells was increased within the tumor‐mimetic microenvironment (Figure 5K,L).

### 2.6 ANGPTL4 Enhances Histone Lactylation Levels to Promote PD‐L1 Expression in HCC Cells

**FIGURE 6:**
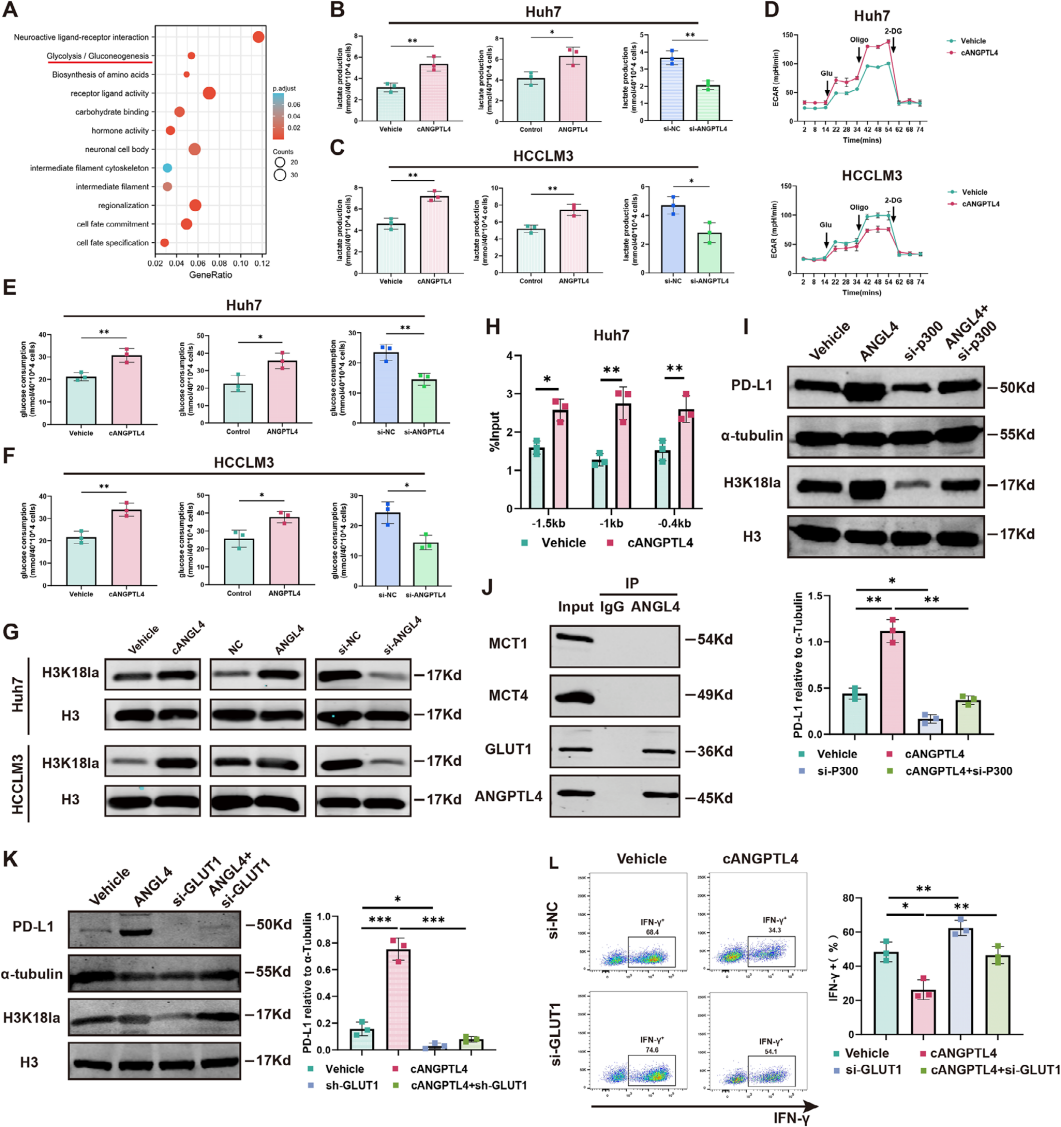
ANGPTL4 increases histone lactylation levels to promote PD‐L1 expression in HCC cells. (A) Pathways related to ANGPTL4 in the GEO database. (B,C) Lactic acid production in Huh7 (B) and HCCLM3 (C) cells treated with cANGPTL4 or conditioned media from CAFs transfected with si‐ANGPTL4 or ANGPTL4 plasmid. (D) A Seahorse X96 analyzer was used to determine the extracellular acidification rate in HCC cells treated with or without cANGPTL4. E–F Glucose consumption in Huh7 (E) and HCCLM3 (F) cells under similar treatments. (G) Effect of ANGPTL4 on H3K18la levels. (H) ChIP analysis of H3K18la enrichment at the PD‐L1 promoter. (I) Western blot analysis of PD‐L1 expression in Huh7 cells treated with cANGPTL4 and/or si‐P300. (J) Co‐IP analysis of ANGPTL4 binding to GLUT1, MCT1, and MCT4. (K) PD‐L1 protein levels in Huh7 cells treated with cANGPTL4 and/or shGLUT1. (L) IFN‐γ levels in CD8^+^ T cells under panel K conditions. (n = 3; data are presented as the mean ± SD; ^*^ *p* < 0.05, ^**^ *p* < 0.01, ^***^ *p*<0.001.).

Bioinformatic analysis of GEO datasets revealed the association of ANGPTL4 with glycolytic pathways in patients with HCC (Figure 6A). Given the established role of aerobic glycolysis and lactate metabolism in tumor progression, we quantified these metabolic fluxes. ANGPTL4 expression significantly increased aerobic glycolysis in HCC cells, as evidenced by increased glucose consumption and lactate production, which were abrogated when CAF‐derived ANGPTL4 secretion was inhibited (Figure 6B–F). Consistent with these results, cANGPTL4 supplementation was shown to increase glycolytic activity in HCC patient‐derived organoids (PDOs) (Figure S7A,B).

Because lactate‐derived histone lactylation, particularly H3K18la, represents an emerging epigenetic mechanism, we hypothesized that ANGPTL4‐mediated lactate accumulation might regulate this modification. Validation experiments confirmed that ANGPTL4 increased H3K18la levels in HCC cells, whereas inhibiting ANGPTL4 secretion from CAFs suppressed H3K18la levels in HCC cells (Figure 6G). Furthermore, we also examined other known histone modification sites, including the lactylation sites H3K9la and H3K23la and the acetylation site H3K18ac. The results revealed that cANGPTL4 had no effect on H3K18ac or H3K23la and only slightly upregulated H3K9la (Figure S7C,D). Therefore, we subsequently focused our research on H3K18la modification. Chromatin immunoprecipitation (ChIP) revealed that cANGPTL4 treatment increased H3K18la enrichment specifically at the PD‐L1 promoter region (Figure 6H). Given that p300 plays an established role as the primary writer enzyme for H3K18la, we assessed PD‐L1 regulation via p300 depletion. siRNA‐mediated p300 knockdown not only reduced basal PD‐L1 expression but also reversed ANGPTL4‐induced upregulation of PD‐L1 expression (Figure 6I; Figure S7E). To explore glycolytic activation mechanisms, we examined lactate transporters (MCT1/4) and the glucose transporter GLUT1. Co‐immunoprecipitation confirmed that ANGPTL4 specifically interacted with GLUT1 but did not bind to MCT1 or MCT4 (Figure 6J; Figure S7F). To elucidate the specific regulatory mechanism of ANGPTL4 on GLUT1, we examined the changes in GLUT1 subcellular localization, protein stability, and function following cANGPTL4 treatment. The results revealed that cANGPTL4 treatment did not affect GLUT1 expression levels on the Huh7 cell membrane (Figure S8A) or its protein degradation rate (Figure S8C); however, it significantly increased cellular glucose consumption (Figure S8B). These findings indicate that the binding of ANGPTL4 to GLUT1 does not affect its localization or stability but rather functions by increasing its transport efficiency.

Rescue experiments confirmed that GLUT1 knockdown abrogated ANGPTL4‐induced upregulation of PD‐L1 expression and H3K18la elevation (Figure 6K; Figure S7G). Flow cytometry further demonstrated that the cytotoxic activity of CD8^+^ T cells was restored upon GLUT1 depletion (Figure 6L). Colony formation assays confirmed that GLUT1 knockdown reversed ANGPTL4‐driven HCC proliferation (Figure S9A,B). To evaluate therapeutic synergy, we inhibited the NNMT–ANGPTL4 axis in CAFs and treated HCC cells with the resulting conditioned media supplemented with PBMCs and anti‐PD‐L1 antibodies. Both CCK‐8 and clonogenic assays revealed that NNMT‐ANGPTL4 axis blockade significantly potentiated the efficacy of anti‐PD‐L1 therapy against HCC cells (Figure S9C–F).

### 2.7 ANGPTL4 Promotes Angiogenesis in HCC Cells

**FIGURE 7:**
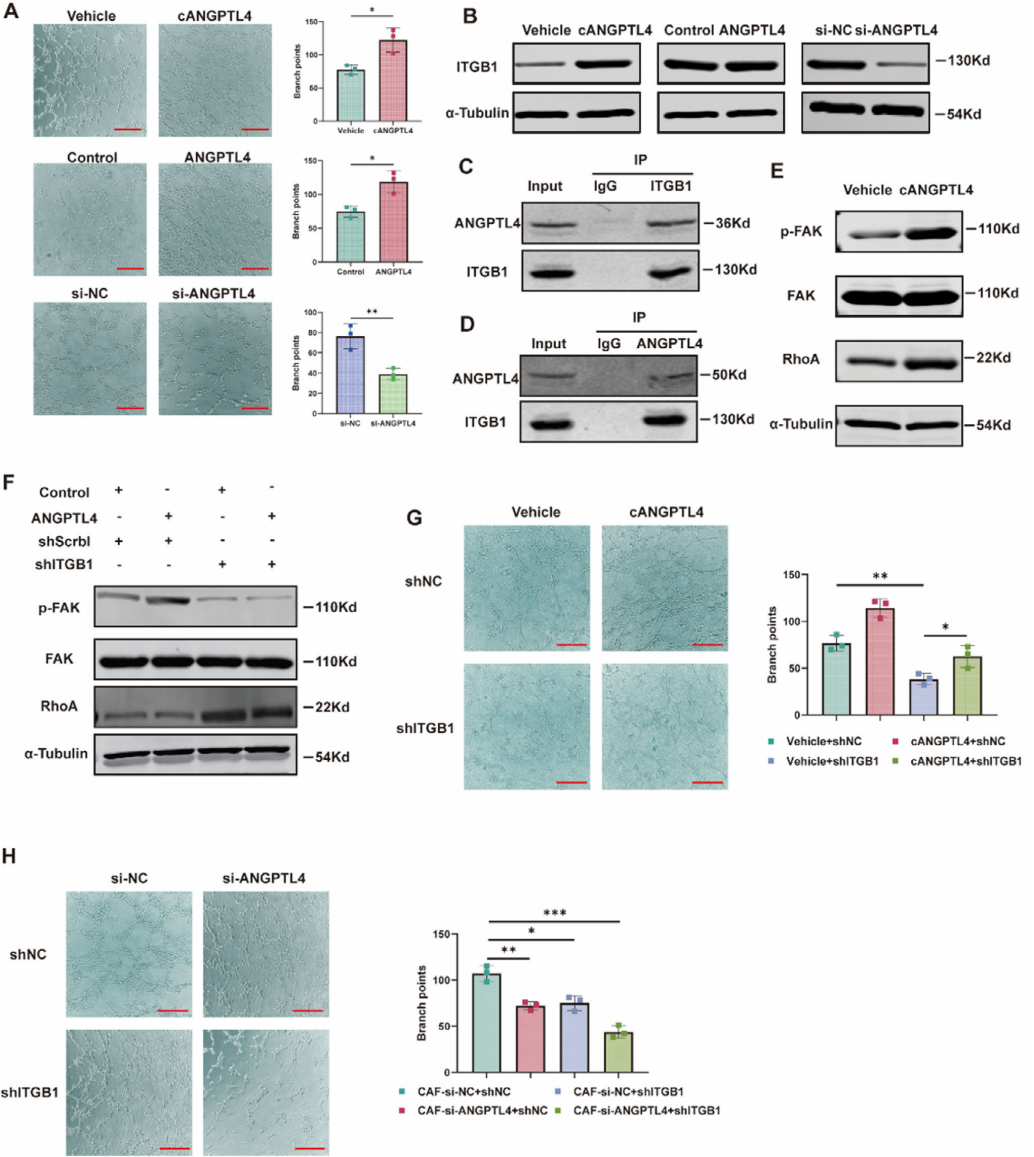
ANGPTL4 promotes angiogenesis in HCC. (A) Representative image of the ability of ANGPTL4 to form vascular‐like tubular structures. (B) Effect of ANGPTL4 on ITGB1 protein levels. (C,D) Co‐IP analysis of the binding between ANGPTL4 and ITGB1. (E) The expression levels of FAK–RhoA signaling proteins in HUVECs. (F) WB detection and analysis of FAK–RhoA signaling protein levels in HUVECs treated with cANGPTL4 and/or shITGB1 in the culture medium. (G) Representative image of the tube formation assays of HUVECs following shITGB1 transfection and/or cANGPTL4 treatment. (H) Representative image of the tube formation assays of HUVECs following treatment with shITGB1 and/or supernatant from ANGPTL4‐knockdown CAFs. (n = 3; data are presented as the mean ± SD; ^*^ *p* < 0.05, ^**^ *p* < 0.01, ^***^ *p* <0.001.).

ANGPTL4 is a member of the angiopoietin family that is structurally similar to angiopoietins and plays a significant role in angiogenesis [14, 15]. However, whether ANGPTL4 regulates angiogenesis in patients with HCC remains unknown. The results demonstrated that ANGPTL4 promotes angiogenesis in vitro. ANGPTL4 knockdown in CAFs weakened their ability to promote angiogenesis in conditioned medium (Figure 7A). It has been reported that ANGPTL4 exerts its biological function by affecting ITGB1, an important membrane protein of vascular endothelial cells [16, 17]. Our results revealed that ANGPTL4 can promote the expression of ITGB1 on the surface of vascular endothelial cells and that these proteins can bind together (Figure 7B–D). ANGPTL4 also activated the FAK–RhoA signaling pathway, which is a classical downstream pathway of ITGB1 (Figure 7E,F). Tube formation assays revealed that ITGB1 knockdown inhibited the angiogenesis of cANGPTL4 (Figure 7G). Furthermore, inhibiting the expression of ANGPTL4 in CAFs and ITGB1 in endothelial cells significantly inhibited angiogenesis in HCC (Figure 7H).

### 2.8 The NNMT–ANGPTL4 Axis is a Promising Therapeutic Target for Immunotherapy Treatment for HCC

**FIGURE 8:**
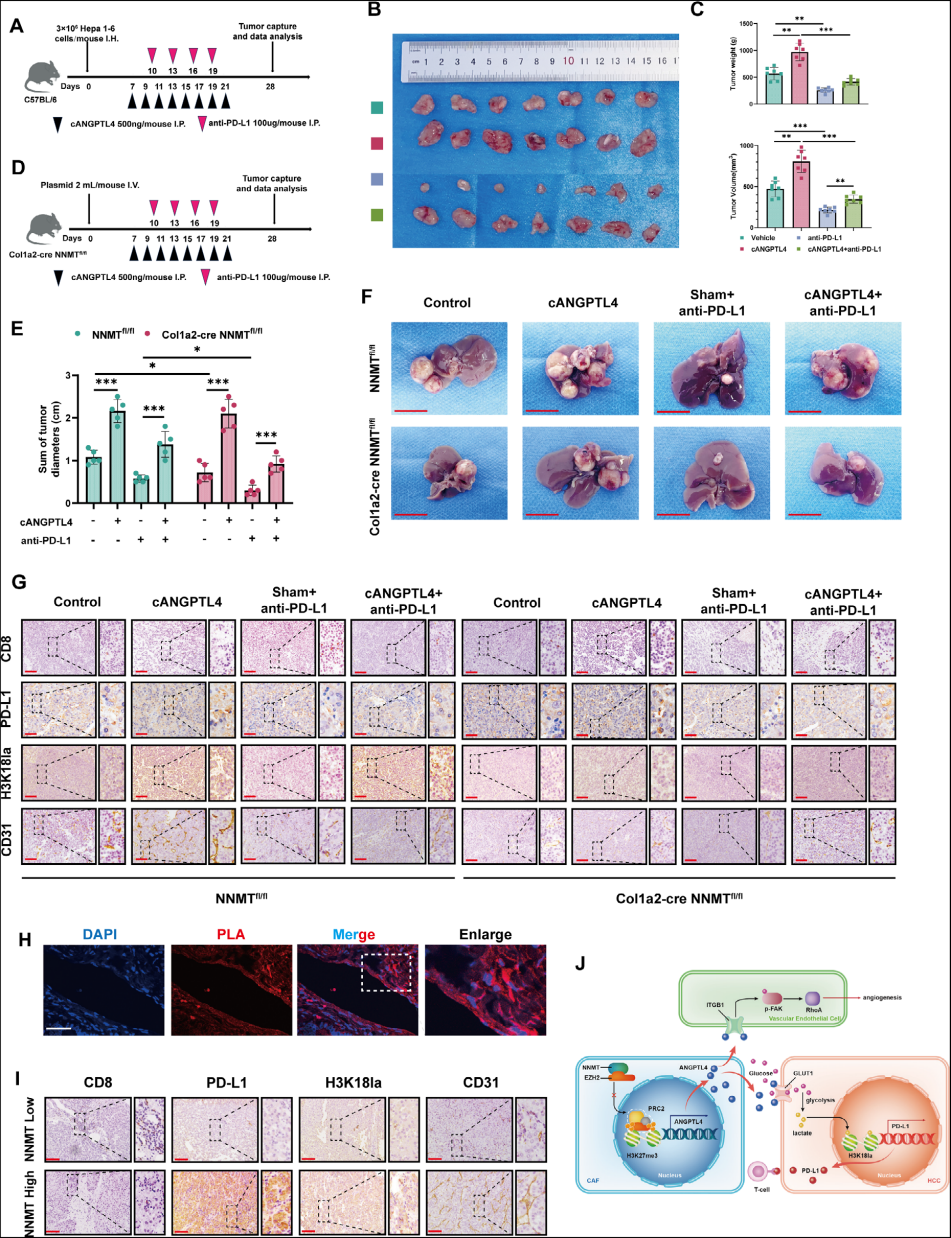
The NNMT‐ANGPTL4 axis is a promising therapeutic target for immunotherapy treatment for HCC. (A) Construction pattern diagram of the C57BL/6 mouse subcutaneous tumor model. (n = 7) (B) Tumor volumes of subcutaneous tumors. (C) Representative images of subcutaneous tumors. (D) Construction pattern diagram of the orthotopic liver tumor models in Col1a2‐Cre NNMT^fl/fl^ mice with specific ablation of NNMT in fibroblasts. (n = 5) (E) Sum of tumor diameters in NNMT^fl/fl^ mice and Col1a2‐Cre NNMT^fl/fl^ mice. (F) Representative images of the orthotopic liver tumor models in NNMT^fl/fl^ mice and Col1a2‐Cre NNMT^fl/fl^ mice. (G) Representative images of IHC staining for CD8, PD‐L1, H3K18la and CD31 in orthotopic liver tumor model tissues. (H) A PLA probe was used to detect the binding of NNMT and EZH2 in orthotopic liver tumor model tissues. (I) Representative images of IHC staining for CD8, PD‐L1, H3K18la and CD31 in human HCC tissues. (J) Schematic of the mechanism through which NNMT promotes ANGPTL4 expression and secretion in CAFs to induce immunosuppressive and angiogenic activities in HCC cells. (Data are presented as the mean ± SD; ^*^ *p* < 0.05, ^**^ *p* < 0.01, ^***^ *p* <0.001.).

In this study, we revealed that the NNMT–ANGPTL4 axis could promote tumor immune escape and angiogenesis in vitro. Therefore, we evaluated whether inhibiting the NNMT–ANGPTL4 axis could increase the efficacy of anti‐PD‐L1 therapy in vivo. Hepa 1–6 cells were injected into C57BL/6 mice to establish subcutaneous tumors. Starting on day 7, the mice received an intraperitoneal injection of recombinant cANGPTL4 every 2 days for a total duration of 14 days. Starting from day 10, the anti‐PD‐L1 antibody was injected into the abdominal cavity of the mice every 3 days for a total of 4 doses (Figure 8A). The results showed that intraperitoneal administration of cANGPTL4 significantly increased tumor volume. The anti‐PD‐L1 antibody markedly suppressed tumor progression induced by cANGPTL4 injection (Figure 8B,C). To further validate the critical role of NNMT in CAFs, we constructed fibroblast‐specific NNMT knockout transgenic mice (Figure S10A,B) and a spontaneous liver tumor model and found that liver tumor size was significantly smaller in the knockout group than in the control group. Additionally, the response to combined anti‐PD‐L1 antibody treatment improved in the knockout group, whereas intraperitoneal injection of cANGPTL4 weakened the anti‐PD‐L1 effect (Figure 8D–F; Figure S10C–F). IHC staining assays revealed that after NNMT was specifically knocked out in fibroblasts, the expression of PD‐L1, CD31, and H3K18la in tumor tissues significantly decreased, whereas CD8^+^ T cells significantly increased. Exogenous injection of cANGPTL4 could compensate for this phenomenon to a certain extent (Figure 8G).

Next, we isolated 8 human HCC tissue samples to construct patient‐derived xenograft (PDX) models and divided them into NNMT‐high and NNMT‐low groups on the basis of their NNMT expression levels in CAFs. These PDX models were transplanted subcutaneously into NOG mice to generate tumors, and PBMCs were injected into the tail vein. ANGPTL4‐ASO was injected into the tail vein to inhibit ANGPTL4 expression in mice. In the NNMT‐low group, ANGPTL4‐ASO did not further increase the therapeutic effect of the anti‐PD‐L1 antibody. However, in the NNMT‐high group, ANGPTL4‐ASO effectively increased the antitumor effects of the anti‐PD‐L1 antibody (Figure S11A–E). Ki‐67 staining of the tumors revealed that inhibiting ANGPTL4 expression significantly restrained tumor proliferation. Moreover, when NNMT expression was low in CAFs, inhibiting ANGPTL4 expression did not lead to significant suppression of tumor growth (Figure S11F,G).

Furthermore, PLA probe assays were conducted on mouse liver cancer tissues, and the results confirmed the interaction between NNMT and EZH2 (Figure 8H). Finally, we validated our results in clinical samples, which showed that the levels of PD‐L1, H3K18la, PD‐L1 and CD31 are positively correlated with NNMT and ANGPTL4 expression in CAFs in HCC tissues (Figure 8I). These results suggest that the NNMT‐ANGPTL4 axis in CAFs may be a promising therapeutic target for HCC. Furthermore, inhibiting ANGPTL4 expression and secretion in the NNMT‐high group could enhance anti‐PD‐L1 efficacy, offering a new direction for HCC immunotherapy.

## 3 Discussion

The TME is a dynamic cell‐to‐cell communication system that plays a crucial role in tumor progression, with CAFs being its important components [18]. CAFs and cancer cells interact through various molecular mechanisms, such as in a paracrine manner [19]. In this study, we found that NNMT promoted ANGPTL4 secretion by CAFs, thereby accelerating PD‐L1 transcription in HCC and promoting immune evasion. The crosstalk between tumor cells and CAFs elucidated the intrinsic mechanisms underlying the clinical progression and immune evasion of HCC.

In our previous studies, we reported that NNMT promotes the proliferation and metastasis of intrahepatic cholangiocarcinoma by inhibiting histone methylation [12]. In 2019, Eckert et al. proposed that NNMT is highly expressed in CAFs and serves as a key metabolic regulator driving tumor progression [9]. Since then, NNMT has been reported to play important roles in gastric cancer and colorectal cancer CAFs [7, 8]. However, the expression profile and biological effects of NNMT in the CAFs of patients with HCC remain unclear. Our findings revealed that NNMT was highly expressed in CAFs and promoted HCC proliferation and immune evasion by increasing ANGPTL4 expression and secretion. Numerous studies have shown that NNMT decreases cellular histone methylation by depleting the universal methyl donor SAM [19, 20]. In this study, we revealed that while NNMT partially decreased SAM levels in CAFs, it did not significantly reduce overall histone methylation but specifically decreased H3K27me3 levels by inhibiting EZH2 nuclear translocation. We speculated that the lower proliferation and metabolic activity of fibroblasts compared with those of parenchymal cells may reduce SAM consumption and dependence. SAM plays crucial roles in histone methylation and various metabolic pathways; thus, the inhibitory effect of NNMT on histone methylation may be weakened [21]. Previous studies have indicated that NNMT overexpression does not significantly alter intracellular SAM levels, suggesting that NNMT inhibits histone methylation not just by depleting SAM [22]. However, exogenous supplementation with SAM partially inhibited ANGPTL4 expression and reversed the biological effects of NNMT, demonstrating that SAM supplementation can compensate for the promoting effect of NNMT on ANGPTL4, although this was not the dominant mechanism involved. On the basis of these findings, we further discovered that the mechanism by which NNMT regulates H3K27me3 levels in CAFs was not through the classical SAM‐dependent pathway but rather through binding to the NLS region of EZH2, thereby inhibiting EZH2 nuclear translocation and subsequently decreasing histone methylation levels. These findings expand the understanding of the molecular mechanisms through which NNMT influences histone methylations within cells.

The role of ANGPTL4 is inconsistently described; it can promote angiogenesis, invasion, and metastasis while mediating chemoresistance through iron metabolism and ferroptosis [23, 24]. Conversely, some studies suggest that it inhibits colon and breast cancer progression [25, 26]. In HCC, most studies suggest that ANGPTL4 promotes tumor progression [27, 28], but some studies also suggest that it has a tumor suppressor effect [29]. Our study revealed that ANGPTL4 derived from CAFs can promote tumor angiogenesis and mediate immune escape in patients with HCC through the promotion of PD‐L1 expression. As an orphan ligand, the specific cellular receptor for ANGPTL4 has not yet been identified [30]. On the basis of its ability to modulate cellular lipid and glucose metabolism, we discovered that ANGPTL4 interacted with GLUT1 on the surface of HCC cells, facilitating glucose transport, activating the aerobic glycolysis pathway, and increasing intracellular lactate levels. Histone lactylation is a newly discovered type of histone modification. In 2019, Zhang et al. first reported histone lactylation, which involved the addition of a lactyl group to lysine residues in the histone tail [31]. Histone lactylation has subsequently been reported in various tumors, such as non‐small cell lung cancer [32]. However, its function in HCC remains unclear. Like other histone modifications, lactylation has been identified as another epigenetic modification involved in the regulation of gene expression. Histone lactylation is sensitive to lactate levels. Decreased lactate production by inhibiting glycolysis reduces lysine lactylation [33]. In this study, we revealed for the first time that ANGPTL4 promoted the transport of glycogen in HCC cells, activated the aerobic glycolysis pathway, and increased cellular lactate accumulation, thereby increasing intracellular histone lactylation levels. Elevated levels of histone lactylation promoted the expression of PD‐L1 and facilitated immune evasion by cancer cells. In endothelial cells, we found that ANGPTL4 bound to the ITGB1 protein and activated the FAK–RhoA signaling pathway to promote angiogenesis, which is consistent with the findings of previous studies [34]. This discovery provides a new target for HCC treatment.

In summary, we propose for the first time that NNMT promotes ANGPTL4 secretion by CAFs, facilitating tumor angiogenesis and immune evasion. Given the significant role of ANGPTL4 in the CAF–HCC interaction, we believe that the NNMT–ANGPTL4 pathway is an important target for increasing immunotherapy responses in patients with HCC.

## 4 Methods

### 4.1 Primary Cell Culture

Primary CAFs and NFs were isolated from HCC and adjacent normal tissues of 30 patients who underwent surgical resection at the First Affiliated Hospital of Harbin Medical University. The patients did not receive chemotherapy or radiation therapy before surgery. Fresh tissues were minced, digested with collagenase VI (2 mg/mL), hyaluronidase (2 mg/mL), and ATP (0.27 mg/mL) at 37°C for 2 h, and cultured in 10 % FBS/DMEM‐F12. The medium was replaced after 48 h to remove debris [34‐35]. The adherent cells were expanded to 1 × 10^6^ cells for 7–10 days. Serum‐starved conditioned media (CM) were collected and stored at −80°C. Subcutaneous injection of 1 × 10^6^ CAFs/NFs into nude mice confirmed no tumor cell contamination (no tumors were observed at 12 weeks). This study was approved by the Ethics Committee of First Affiliated Hospital of Harbin Medical University.

### 4.2 PBMC and CD8^+^ T‐Cells Isolation

Whole blood samples were obtained from healthy donors. Equal volumes of PBS and whole blood were mixed with Ficoll‐Paque media and centrifuged at 400 × *g* for 20 min. After isolating the middle layer of the PBMC cells, the cells were rinsed with PBS and centrifuged at 400 × *g* for 5 min. The supernatant was discarded to obtain the PBMCs. CD8^+^ T cells were isolated from PBMCs using the Dynabeads CD8 Positive Isolation Kit (Invitrogen 11333D).

### 4.3 PLA Assay

PLA assay was performed using Duolink in Situ Orange Starter Kit Mouse/Rabbit (DUO92102, Sigma Aldrich) in HCC cells according to the standard technique. In brief, HCC cells cultured in glass bottom culture dishes were washed with phosphate buffered saline (PBS) solution, fixed with paraformaldehyde for 15 min, permeabilized with 0.5 % Triton X‐100 for 10 min, and then subjected to blocking for 1 h, primary antibody incubation at 4°C overnight, Duolink PLA probe (PLUS and MINUS) incubation for 1 h, ligation reaction for 30 min, PCR amplification for 100 min, and finally imaged under a confocal microscope after the final wash by adding Duolink in situ mounting medium containing DAPI.

### 4.4 Immunohistochemistry (IHC) Staining

IHC staining was performed as reported in our previous study [36]. Briefly, tissue sections were deparaffinized, rehydrated, and then blocked with 10 % normal goat serum. An anti‐NNMT antibody (Aviva Systems Biology, San Diego, CA) (1:200 dilution) or an anti‐Ki‐67 antibody (Cell Signaling Technology, Danvers, MA) (1:500 dilution) was incubated with the sections overnight at 4°C. Sections were then sequentially incubated with a secondary antibody (Vector lab, Burlingame, CA) for 1 h at room temperature and Vectastain Elite ABC reagent (Vector lab) for 30 min. Tissue sections were then stained with diaminobenzidine (DAB kit; Vector Lab) and counterstained with hematoxylin (Sigma). The density of IHC staining was analyzed using Image‐Pro Plus v6.2 software, and the median density values of all the slides with positive staining were used as the cutoff to define high or low expression subgroups.

### 4.5 Immunofluorescence (IF) Staining

IF staining was performed as reported in our previous study [37]. The tissue sections were deparaffinized, rehydrated, and then blocked with 10 % normal goat serum. An anti‐NNMT antibody (Abcam, ab119758) (1:150 dilution) and an anti‐CK‐19 antibody (Proteintech, 10712‐1‐AP) (1:200 dilution) were incubated with the sections overnight at 4°C. The following day, cells were incubated with a fluorescent secondary antibody for 1 h. Finally, nuclei were counterstained with DAPI, and the images were obtained under a confocal laser scanning microscope.

### 4.6 Animal Studies

Four‐week‐old male C57BL/6 mice were purchased from Vital River Laboratory (Beijing, China) and housed under specific pathogen‐free conditions. The animal studies were approved by the Animal Ethics Committee. For the subcutaneous model, 3 × 10^6^ Hepa 1–6 cells suspended in 150 µL of PBS were injected into the flanks of the mice. Tumor size was measured weekly. After four weeks, the tumor was removed, and its weight and volume were recorded.

Four‐week‐old male NOG mice were purchased from Vital River Laboratory (Beijing, China) and housed under specific pathogen‐free conditions. Four‐week‐old male NOG mice were intravenously injected into the tail vein with 5 × 10^6^ PBMCs. The mice were subcutaneously injected with 3 × 10^6^ HCC patient‐derived xenografts in the flank. Measurements of the tumors were carried out every week. At 15 days post‐inoculation, a PD‐L1 mAb or IgG was intraperitoneally injected into the mice every 3 days. After three injections, the tumors were collected for IHC staining.

The plasmid was dissolved in 2 mL of saline and injected into 6‐week‐old male C57BL/6 mice via the tail vein within 5–7 s. We prepared 13 µg of c‐Myc, 10 µg of sgP53, and 8.25 µg of Sleeping Beauty (SB) transposase. Vectors were prepared using the EndoFreeMaxi kit (Qiagen). The survival of the mice and the development of HCC were analyzed.

C57BL/6 Col1a2‐Cre mice were purchased from Bestcell Animal Center (Wuhan, China). C57BL/6 NNMT^flox/flox^ mice were generated as reported in our previous study [37]. By crossing the Nnmt^flox/flox^ mice with Col1a2‐Cre mice, we created a fibroblast‐specific Nnmt‐KO mouse model.

### 4.7 Statistical Analysis

Statistical analysis was performed using GraphPad Prism 9.0 software. Data were tested for normality and homogeneity of variance before formal analysis. Outliers were identified using Grubbs’ test and excluded if appropriate. Data are presented as mean ± standard deviation (SD). Sample size (n) is indicated in each figure legend.

A two‐tailed Student's *t*‐test was used for comparisons between two groups. One‐way or two‐way ANOVA was applied for comparisons among three or more groups, followed by Tukey's HSD post‐hoc test for multiple comparisons. Pearson correlation analysis was performed to evaluate linear relationships. A *p*‐value< 0.05 was considered statistically significant.

Additional details on materials and methods can be found in the Supplemental Materials.

### 4.8 Availability of Data and Materials

All data generated and analyzed during this study are included in this article and its additional file.

## Author Contributions

Shounan Lu and Shanjia Ke designed and performed the experiments, analyzed the data, and wrote the paper. Hongjun Yu, Zhanzhi Meng, and Miaoyu Bai performed experiments and analyzed the data. Yanan Xu, Baolin Qian, Bing Yin, and Chaoqun Wang performed some of the experiments. Zhigang Feng, Zhongyu Li, and Yongzhi Zhou analyzed the data. Zhongyu Li and Yao Fu provided the patient samples for clinical data analysis. Xinglong Li, Yongliang Hua, and Wei Tang provided assistance in the study. Yaohua Wu and Yong Ma initiated the study, organized, designed, and wrote the paper. All authors read and approved the final manuscript.

## Funding

This study was funded by National Natural Science Foundation of China (81502605, 82370643, 82403110, and 82403104), National Outstanding Youth Science Fund Project of National Natural Science Foundation of China (82525068), Scientific Foundation of the First Affiliated Hospital of Harbin Medical University (HYD2020JQ0007 and HYD2020JQ0012), Chen Xiaoping Foundation for the Development of Science and Technology of Hubei Province (CXPJJH122002‐025), Key Research and Development Program of Heilongjiang Province (2024ZX12C28), Natural Science Foundation of Heilongjiang Province of China (LC2018037 and PL2024H069), Medical and Health Research Project of Zhejiang Province (2024KY1324), Basic scientific research projects of provincial higher education institutions in Heilongjiang Province (2023‐KYYWF‐0151).

## Ethics Approval and Consent to Participate

This study was examined and certified by the Ethics Committee of First Affiliated Hospital of Harbin Medical University (2022124). The informed consent was obtained from all participants included in the study, in agreement with institutional guidelines.

## Conflicts of Interest

The authors declare no conflicts of interest.

## Supporting information




**Supporting File**: advs75181‐sup‐0001‐SuppMat.pdf.

## Data Availability

The data that support the findings of this study are openly available in GEO at https://www.ncbi.nlm.nih.gov/geo/query/acc.cgi?acc=GSE149614, reference number 149614. These data were derived from the following resources available in the public domain:[GSE149614], https://www.ncbi.nlm.nih.gov/geo/query/acc.cgi?acc=GSE149614 [GSE146115], https://www.ncbi.nlm.nih.gov/geo/query/acc.cgi?acc=GSE146115 [GSE151530], https://www.ncbi.nlm.nih.gov/geo/query/acc.cgi?acc=GSE151530 [GSE189903], https://www.ncbi.nlm.nih.gov/geo/query/acc.cgi?acc=GSE189903.
