# Supplementary material for: Cancer‐Associated Fibroblasts Promote Tumor Immunosuppression in Hepatocellular Carcinoma via the NNMT‐ANGPTL4 Axis
Source: Adv Sci (Weinh). 2026 Apr 14:e21418. Online ahead of print. doi: 10.1002/advs.202521418 (PMC13334667; doi:10.1002/advs.202521418)
Supplement: Supplementary file 1 — Supporting File: advs75181‐sup‐0001‐SuppMat.pdf. [file ADVS-9999-e21418-s001.pdf]

Cancer Associated Fibroblasts Promote Tumor Immunosuppression in Hepatocellular Carcinoma via the NNMT-ANGPTL4 Axis

Shounan LU[1,2]*, Shanjia KE[2,3]*, Hongjun YU[1,2]*, Zhanzhi MENG[1,2]*, Miaoyu BAI[1,2,9]*, Yanan XU[2,4*], Hui Zhu[1,2], Jinwen Yang[1], Baolin QIAN[1,2], Bing YIN[1,2], Chaoqun WANG[2,5], Zhigang FENG[2,6], Zhongyu LI[1,2], Yongzhi ZHOU[1,2], Zihao LI[1,2], Xinglong LI[1,2], Yongliang HUA[2,7], Yao FU[8], Wei TANG[10,11], Yaohua WU[2,12#], Yong MA[1,2#]

Supplementary methods

Clinical samples and cell lines

The tissues were collected from 30 patients with HCC who underwent liver resection at the First Affiliated Hospital of Harbin Medical University between August 2018 and September 2020. Patients with unresectable hepatocellular carcinoma, as confirmed by multidisciplinary team evaluation, received atezolizumab monotherapy. The initial efficacy assessment was performed 2 months (±1 week) after treatment initiation according to the modified Response Evaluation Criteria in Solid Tumors (mRECIST). Patients achieving complete response (CR) or partial response (PR) were defined as the treatment-sensitive group, while those with progressive disease (PD) were defined as the treatment-insensitive group. Baseline serum samples were collected at the patients first visi t. All participants provided written informed consent. This study was approved by the Research Ethics Committee of the First Affiliated Hospital of Harbin Medical University. The Chinese Academy of Science (Shanghai, China) provided Huh7, HCCLM3 and Huvec cell lines. DMEM with 10% fetal bovine serum was used as the cell culture medium.

All the cell lines were cultured at 37 °C in 5% CO_2. All cell lines were authenticated by the suppliers.

RNA extraction and qRT-PCR

Total RNA was extracted from cells and tissues using an RNA Miniprep Kit

(Axygen), quantified with a Nanodrop ND-2000 spectrophotometer and reverse transcribed into cDNAs using a High Capacity Reverse Transcription Kit (Applied Biosystems). Real-time PCR was performed using Power SYBR Green PCR Master Mix (Applied Biosystems) and an ABIPRISM 7500HT instrument (Applied Biosystems). The expression levels of the indicated mRNAs were determined using the ΔΔCt method with beta-actin as an internal control. A complete list of primer sets is provided in Table S1.

Western blotting

Tissues and cells were lysed with RIPA buffer, and proteins were harvested. Forty micrograms of protein were separated on SDS–PAGE gels and transferred to PVDF membranes (Invitrogen). Detailed descriptions of the antibodies are provided in Table S2. Proteins were visualized and quantified using an Odyssey CLx Imaging System (LI-COR Biosciences, USA).

Cell transfection

Lenti-shNNMT (5′-CATGATTGGTGAGCAGAAG-3′) and the corresponding control vectors were designed and purchased from GeneChem (Shanghai, China) in our previous study [1]. Lenti-NNMT, lenti-shGLUT1(5′-CTTCTATTACTCCACGAG CAT-3′), lenti-shITGB1 (5′-CCTCCAGATGACATAGAAA-3′) and the corresponding control vectors were designed and purchased from HanBio (Shanghai, China). Following transfection, successfully transfected cells were selected in the presence of puromycin (Sigma–Aldrich Corp., St. Louis, MO, USA) for 2 weeks. For siRNA and plasmid transfection, Lipofectamine 2000 reagent was used according to the manufacturer's protocol.

CCK-8 assay

For this assay, 500-1000 cells were seeded in each well of a 96-well plate. The optical density (OD) value was determined by adding the CCK-8 (Dojindo, Japan) solution at the indicated time points and incubating the cells for two hours at 37 °C.

Colony formation assays

For colony formation assays, 500-1000 cells were seeded into each well of 6-well

plates and cultured for 14 days. Then, the colonies were fixed with 4% paraformaldehyde (PFA) and stained with 0.5% crystal violet to visualize colonies

EdU assay

An EdU analysis kit (Beyotime, China) was used to assess DNA synthesis and cell proliferation. A total of 100,000 treated HCC cells were seeded in each well of a 24-well plate and cultured overnight. The next day, an EdU solution (10 μM) was added to each well of the 24-well plate and incubated for 2 h. Next, 4% formalin was added, and the HCC cells were fixed at room temperature for 2 h. In the next step, the HCC cells were then infiltrated with 0.5% Triton X-100 for 10 min, Click Additive Solution (200 μL) was added to stain the EdU for 30 min, and Hoechst 33342 (200 μL) was added to stain the nucleus. Finally, a Nikon microscope (Nikon, Japan) was used to observe DNA synthesis and cell proliferation, which were reflected by the red and blue signals, respectively.

Coimmunoprecipitation assay

Total proteins were extracted with cell lysis buffer (Cell Signaling Technology, Danvers, MA) supplemented with protease inhibitor and phosphatase inhibitor (Cell Signaling Technology, Danvers, MA). The lysate was incubated with anti-NNMT (Aviva), anti-EZH2 (Cell Signaling Technology, Danvers, MA), anti-ANGPTL4, anti-GLUT1 and IgG antibodies (Cell Signaling Technology, Danvers, MA) (as a negative control) at 4 °C overnight with gentle rotation. Then, the protein-antibody complexes were incubated with Protein A/G Plus Agarose (Santa Cruz, USA) for 5 h at 4 °C with gentle rotation. Immunoprecipitates were then collected by centrifugation at 14000×g for 30 s at 4 °C, after which the bead complexes were washed three times with cell lysis buffer. After the final wash, proteins were eluted from Protein A/G agarose by boiling in 5X SDS sample buffer at 100 °C for 5 mins before western blotting analyses.

Chromatin Immunoprecipitation (CHIP)

Formaldehyde fixation, cell lysis, and sonication were conducted as previously described. In total, 1 μg anti-H3K18la, 1 μg anti-H3K27me3, 1 μg anti-EZH2, 1 μg H3K18la or 1 μg nonspecific immunoglobulin G (Santa Cruz) was used to immunoprecipitate the chromatin. Input and immunoprecipitated DNA were subjected

to reversal of the cross-links and purification followed by real-time PCR. A complete list of primer sets is provided in Supplementary Table 1. The product was run on a 5% polyacrylamide gel. Electrophoresis results were quantified using PhosphoImager (Molecular Dynamics) and Image Quant software.

ELISA

Cells were counted and plated in 96-well plates and cultured for 24 h. The supernatants were subsequently collected, and 0.45 mm filters were used to eliminate floating cells. The amount of IFN-γ and TNF-α protein in the supernatant was determined using human IFN-γ- and TNF-α-specific ELISA kits, respectively. All the samples were evaluated in biological replicates and technical triplicates.

ATP concentrations, glucose consumption and lactate secretion

Adenosine triphosphate (ATP) concentrations, glucose consumption and lactate secretion were measured as described in our previous report. ATP concentrations were quantified with an ATP Determination Kit (Beyotime, China) using a VarioSkan flash fluorescence plate reader (Thermo Scientific, USA) according to the manufacturer's instructions. Glucose levels in the culture medium were measured using an assay kit from Nanjing Jiancheng Bioengineering Institute (Nanjing, China). The lactate level in the culture medium was detected using a lactate assay kit (Biovision Inc., USA). All data were normalized by cell number.

Extracellular acidification rate (ECAR)

The extracellular acidification rate (ECAR) of the HCC cells was measured using a Seahorse XF96 flux analyzer (Seahorse Bioscience, Billerica, Massachusetts, USA) according to the manufacturers instructions. The data were normalized to the total protein content.

Determination of SAM and SAH levels

SAM and SAH levels were determined as reported in our previous study [1]. The concentrations of SAM and SAH were determined using reversed-phase high-performance liquid chromatography (HPLC). The cells ($1x10^7$) were centrifuged, washed twice with PBS, and maintained on ice. The cell pellets were subsequently homogenized in 100 μL of 0.4 M ice-cold perchloric acid. The homogenates were

subsequently centrifuged at 15,000 × g for 15 min at 4 °C. The supernatants were collected and stored at -80 °C until analysis. The supernatant of each sample was loaded on a C18 column (SunFire, Ireland) and analyzed using a Waters HPLC system (Milford, MA). The two mobile phases were as follows: mobile phase A consisted of 50 mM NaH_2PO_4 and 8 mM heptanesulfonic acid (pH=3.0), and mobile phase B contained methanol. Isocratic elution was achieved at a flow rate of 1 mL/min with the following parameters: 80% mobile phase A and 20% mobile phase B. The total run time was 15 min, and the injection volume was 20 μL. The absorbance of the analytes was recorded by a UV detector at 254 nm. SAM and SAH standards purchased from Sigma–Aldrich (St. Louis, MO, USA) were used to identify the elution peaks, and the cellular SAM and SAH levels were calculated using automatic peak area integration.

Patient-derived organoid (PDO) models

PDO concentrations were established with a PDO Model Kit (Absin). Briefly, each HCC sample was minced on ice and enzymatically digested for 0.5–2 h at 37 °C on an orbital shaker. The incubation continued until the digestion preparation was visually inspected. Thereafter, the suspension was strained through a 75-μm nylon filter. The supernatant was removed, and the pellet was washed in precooled phosphate-buffered saline (PBS), mixed with a Matrigel matrix (Absin), and seeded in a 24-well or 96-well plate. After polymerization of the GFR Matrigel matrix, PDO culture medium (Absin) was added. The culture medium was changed twice per week. The PDOs were passaged every 1–2 weeks after dissociation with dispase. For storage, the PDOs were dissociated, resuspended in recovery cell culture freezing medium (Stem Cell Technologies), and frozen using standard procedures.

Reference

[1] Lu S, Ke S, Wang C, et al. NNMT promotes the progression of intrahepatic cholangiocarcinoma by regulating aerobic glycolysis via the EGFR-STAT3 axis. *Oncogenesis*. 2022;11(1):39.

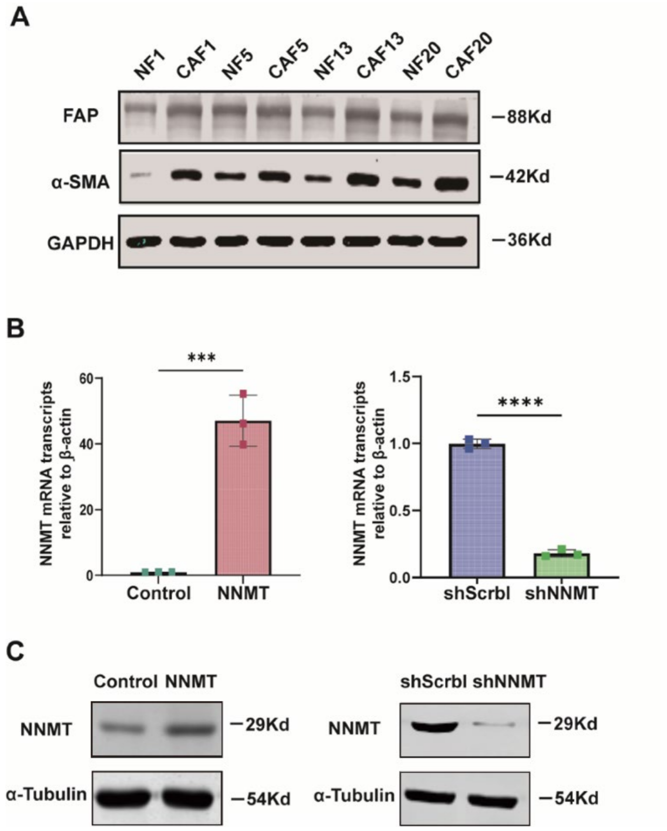

Supplementary Figure 1

Expression of NNMT and CAF markers in fibroblasts and the transfection efficiency of NNMT in CAFs.

A Expression of NNMT and CAF markers (FAP and α-SMA) in fibroblasts. **B** qRT–PCR was used to analyze NNMT expression in CAFs transfected with NNMT-overexpressing lentiviral vectors or NNMT-knockdown lentiviral vectors and their related control vectors. **C** Western blotting was used to analyze NNMT expression in CAFs transfected with NNMT-overexpressing lentiviral vectors or NNMT-knockdown lentiviral vectors and their related control vectors. (n=3; data are presented as the mean ± SD; *P < 0.05, **P < 0.01, ***P<0.001.)

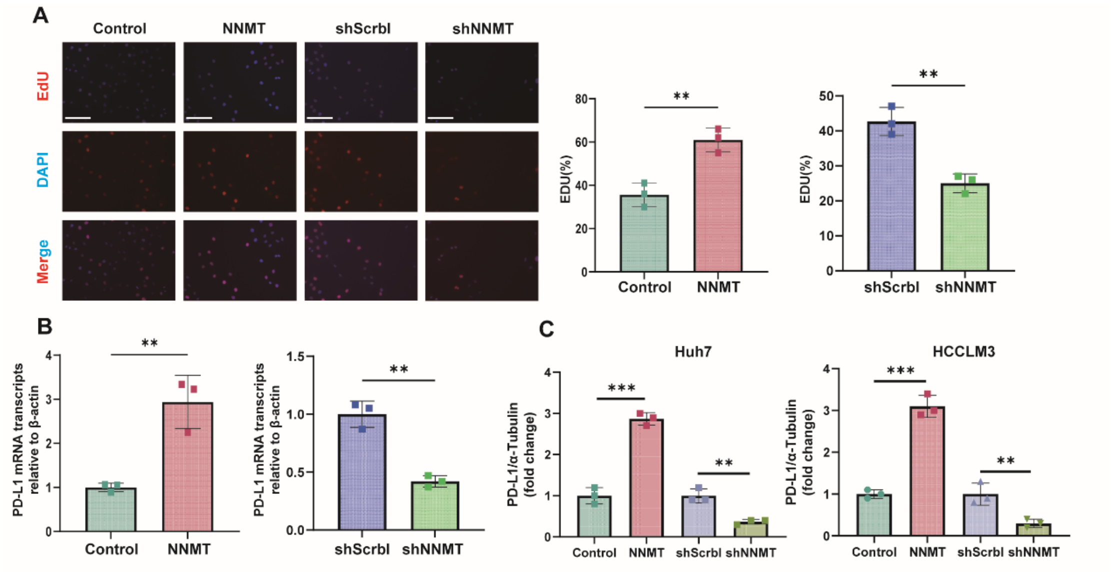

NNMT overexpression in CAFs promotes HCC cell proliferation and PD-L1 expression.

A Representative image of the EdU proliferation experiment Huh7 and HCCLM3 cells following shNNMT- or NNMT-overexpressing after NNMT-overexpressing or –knockdown cells and the control CAFs supernatant treatment. **B** qRT–PCR was used to analyze PD-L1 expression in CAFs transfected with NNMT-overexpressing lentiviral vectors or NNMT-knockdown lentiviral vectors and their related control vectors. **C** Western blotting was used to analyze PD-L1 expression in CAFs transfected with NNMT-overexpressing lentiviral vectors or NNMT-knockdown lentiviral vectors and their related control vectors. (n=3; data are presented as the mean ± SD; *P < 0.05, **P < 0.01, ***P<0.001.)

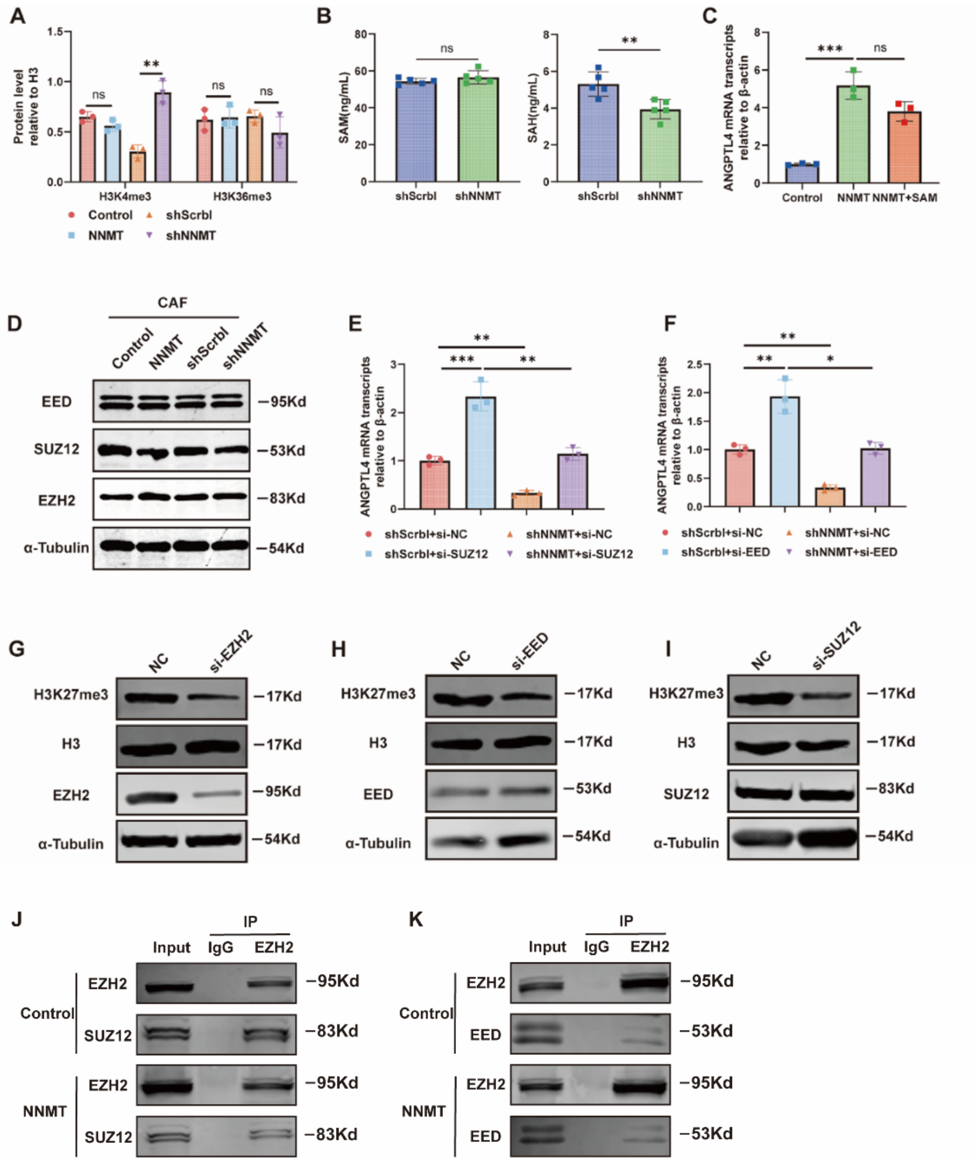

Supplementary Figure 3

NNMT and the PRC2 complex jointly regulate H3K27me3 levels and promote ANGPTL4 expression.

A WB analysis of the effect of NNMT on histone modifications. **B** HPLC was used to measure the levels of SAM and SAH in CAFs. **C** qPCR analysis of the ANGPTL4 mRNA level in CAFs treated with SAM in the culture medium. **D** Western blot analysis of the effect of NNMT on PRC2 complex levels in CAFs. **E–F** qPCR analysis of ANGPTL4 mRNA in CAFs transfected with shNNMT and/or si-SUZ12 **(E)** or si-EED **(F)**. **G–I** Effects of si-EZH2 **(G)**, si-EED **(H)**, and si-SUZ12 **(I)** on H3K27me3 levels in CAFs. **J–K** The binding of EZH2 with SUZ12 (**J**) and EED (**K**) after NNMT overexpression. (n=3; data are presented as the mean ± SD; *P < 0.05, **P < 0.01, ***P<0.001.)

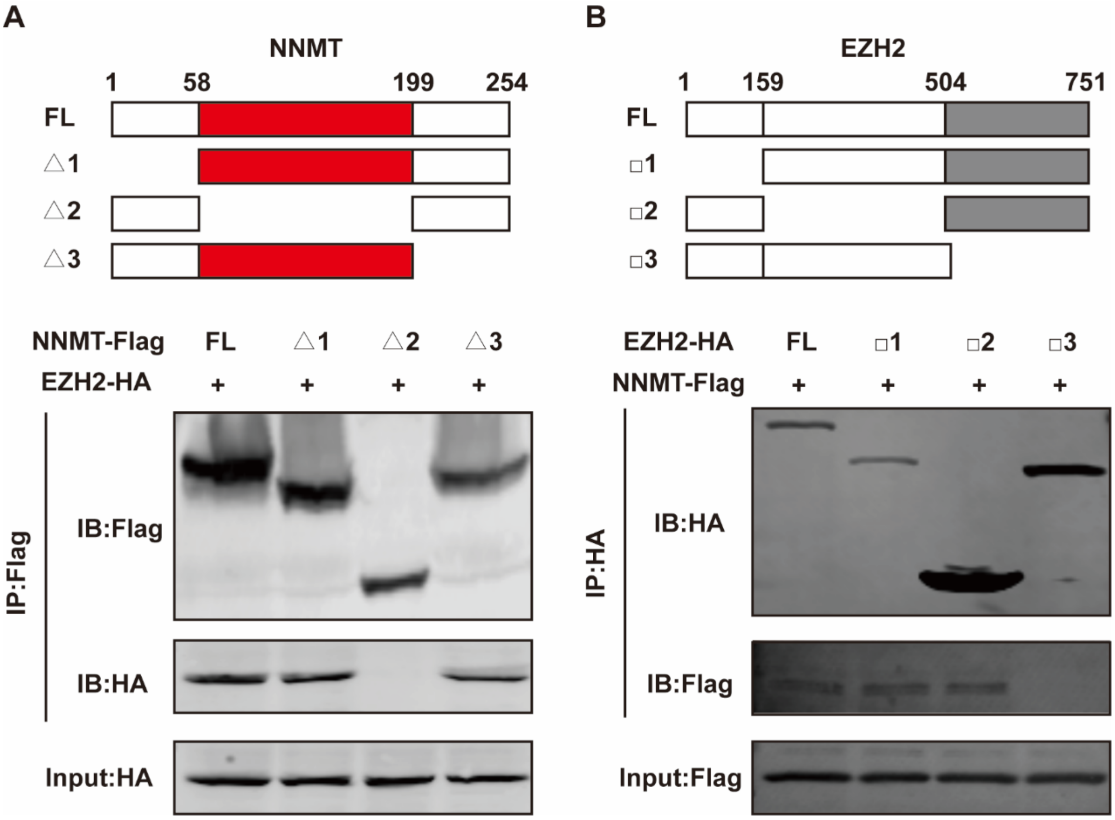

Supplementary Figure 4

The critical domain for the binding of NNMT to EZH2.

A Schematic representation of full-length NNMT and different truncation mutants of NNMT (upper panel). Full-length or indicated truncation mutants of NNMT-Flag were coexpressed with EZH2-HA in HEK293T cells. NNMT-Flag was immunoprecipitated with an anti-Flag antibody, followed by immunoblotting analysis of EZH2-HA using an anti-HA antibody (lower panel). **B** Schematic representation of full-length EZH2 and different truncation mutants of EZH2 (upper panel). Full-length EZH2-HA or indicated truncation mutants of EZH2-HA were co-expressed with NNMT-Flag in HEK293T cells. EZH2-HA was immunoprecipitated with an anti-HA antibody, followed by immunoblotting analysis of NNMT-Flag using an anti-Flag antibody (lower panel).

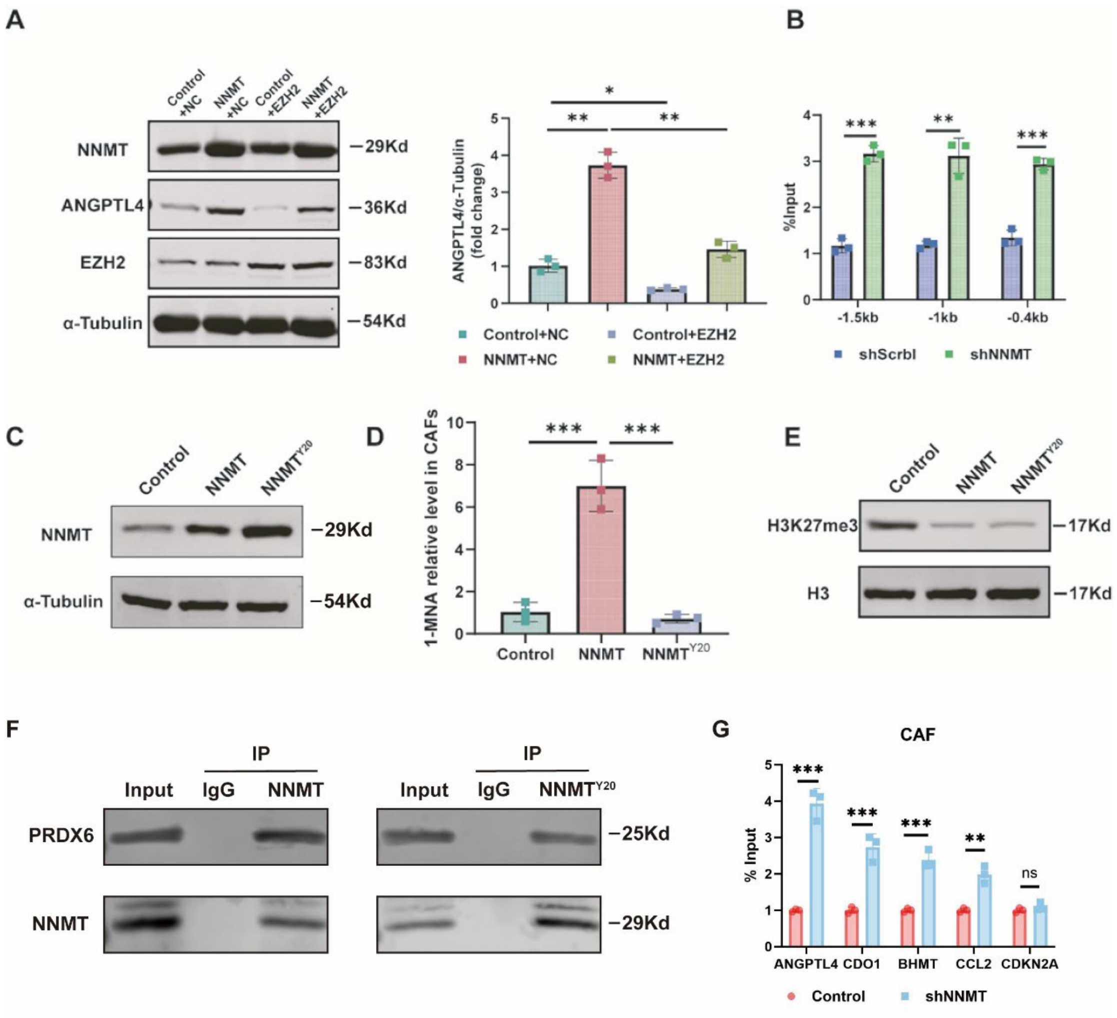

Supplementary Figure 5

NNMT suppresses H3K27me3 levels independently of its methyltransferase activity.

A Effects of EZH2 and NNMT on ANGPTL4 protein levels. **B** ChIP analyses of the degree of enrichment of EZH2 at the ANGPTL4 promoter region. **C** WB analysis of the effects of NNMT and NNMT^{Y20} on the levels of NNMT in CAF. **D** HPLC was used to measure the levels of 1-MNA in CAFs. **E** Effects of the NNMT^{Y20} mutation on H3K27me3 levels. **F**. The NNMT^{Y20} mutant binds to PRDX6. **G** Effect of shNNMT on other known downstream genes of H3K27me3 (CDO1, BHMT, CCL2, and CDKN2A) (n=3; data are presented as the mean ± SD; *P < 0.05, **P < 0.01, and ***P<0.001).

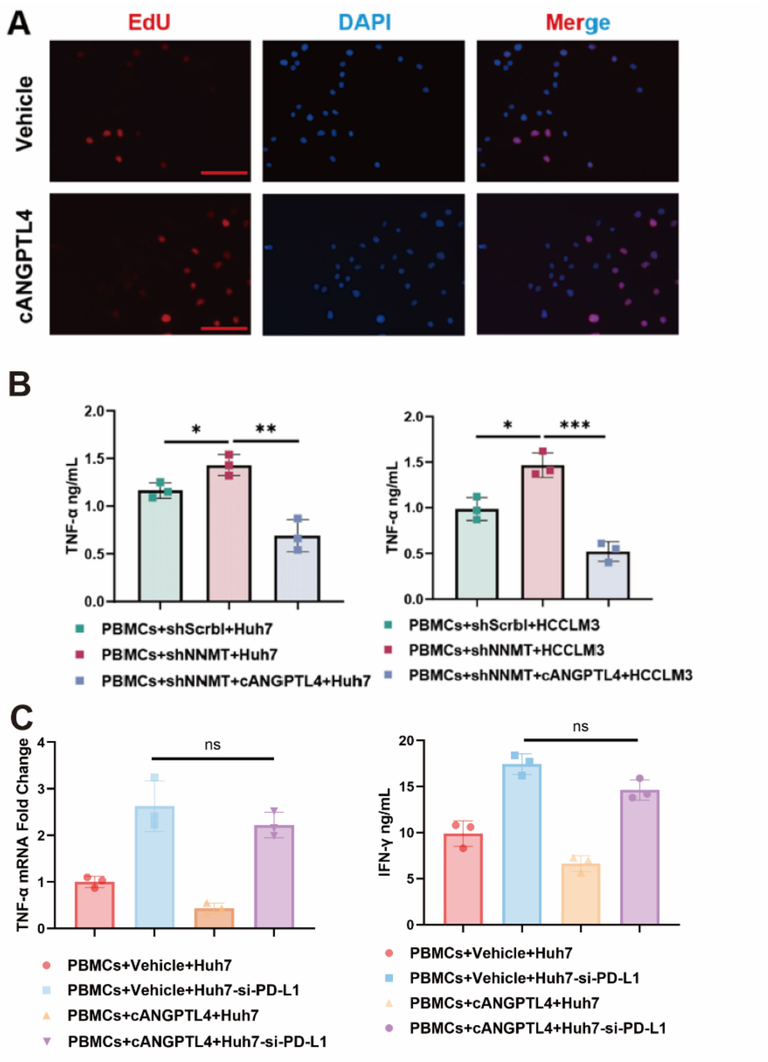

Supplementary Figure 6

ANGPTL4 promotes PD-L1 expression in HCC cells and enhances immune evasion

A Representative image of the EdU proliferation experiment in Huh7 cells following cANGPTL4 treatment. **B** Relative TNF-α mRNA levels in PBMCs treated with Huh7 and HCCLM3 cells following treatment with supernatant from NNMT-knockdown CAFs and cANGPTL4. **C** Relative mRNA levels of TNF-α and IFN-γ in PBMCs treated with Huh7 cells following treatment with si-PD-L1 and cANGPTL4. (n=3; data are presented as the mean ± SD; *P < 0.05, **P < 0.01, ***P<0.001.)

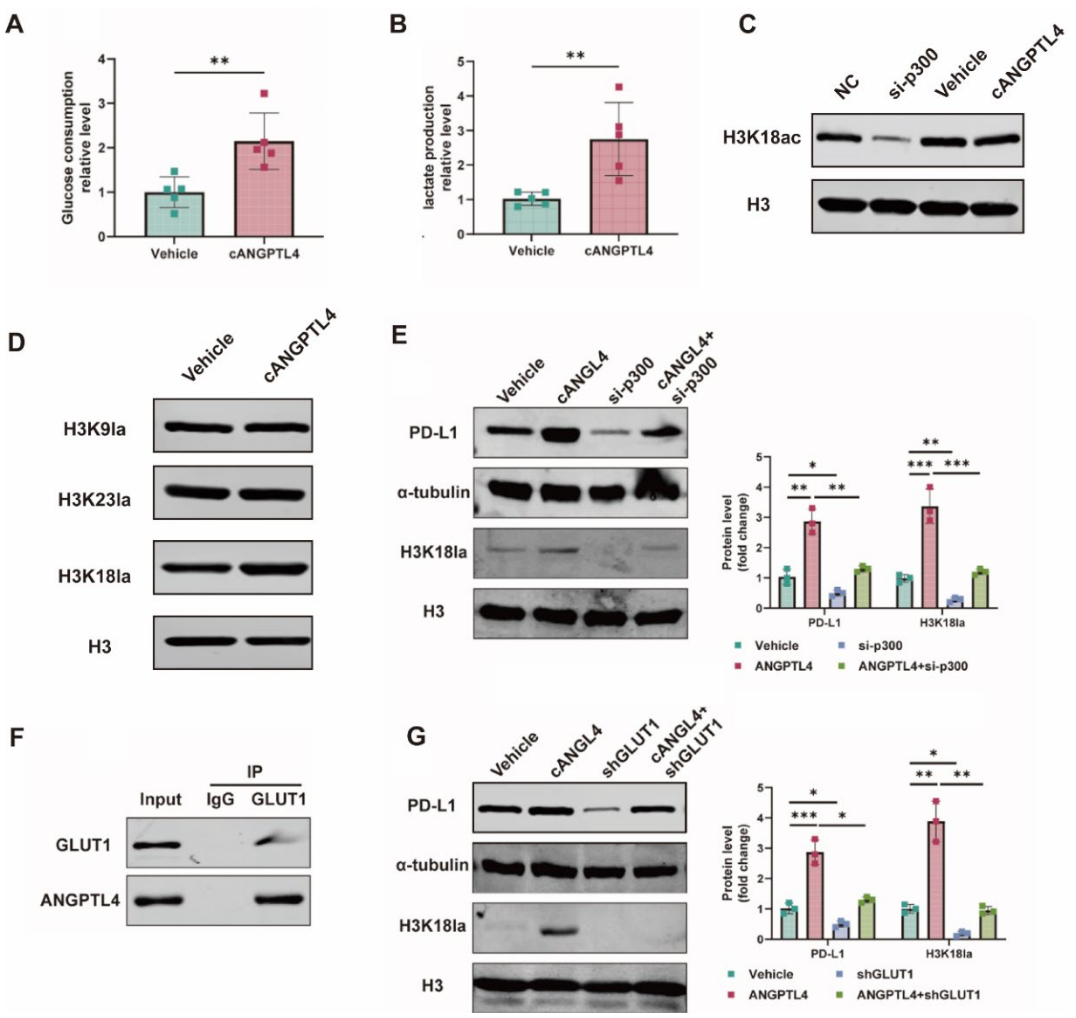

Supplementary Figure 7

ANGPTL4 promotes PD-L1 expression by activating the glycolytic pathway in HCC cells.

A Lactic acid production in the PDO model treated with cANGPTL4. **B** Glucose consumption in the PDO model treated with cANGPTL4. **C** H3K18ac levels in Huh7 cells after treatment with cANGPTL4 or transfection with si-p300. **D** Effect of cANGPTL4 on intracellular H3K9la, H3K18la, and H3K23la levels. **E** WB detection and analysis of the PD-L1 protein level in HCCLM3 cells treated with cANGPTL4 and/or si-P300 in the culture medium. **F** Co-IP analysis of the binding between GLUT1 and ANGPTL4. **G** WB detection and analysis of the PD-L1 protein level in HCCLM3 cells treated with cANGPTL4 and/or shGLUT1 in the culture medium. (n=3; data are presented as the mean ± SD; *P < 0.05, **P < 0.01, ***P<0.001.)

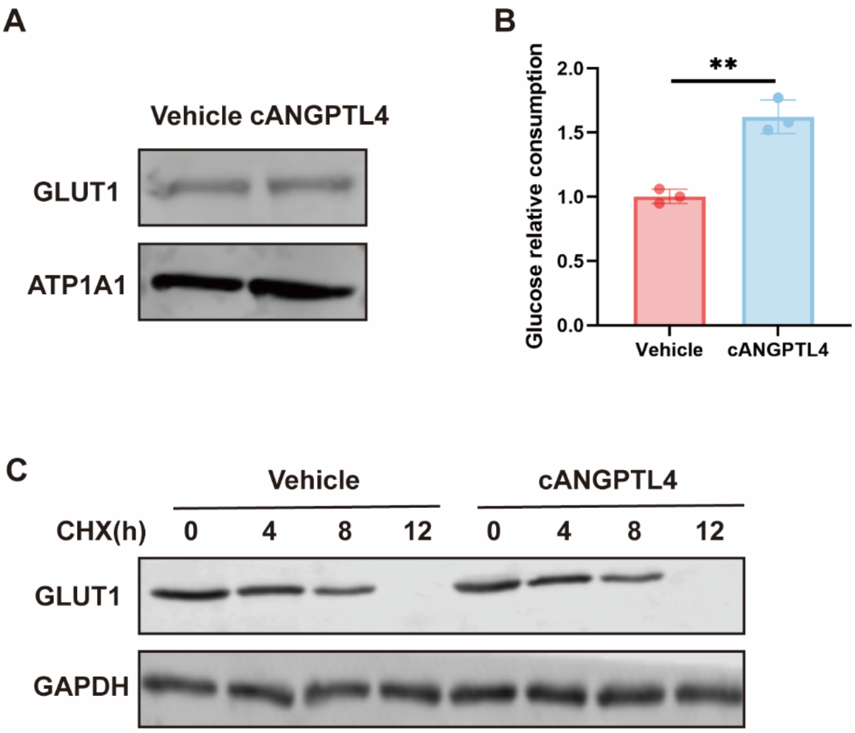

Supplementary Figure 8

ANGPTL4 affects GLUT1 function.

A cANGPTL4 has no effect on GLUT1 expression in Huh7 cells; **B** cANGPTL4 increases glucose consumption in Huh7 cells; **C** cANGPTL4 does not affect GLUT1 protein degradation. (n=3; data are the mean ± SD, $^{**}P < 0.01$.)

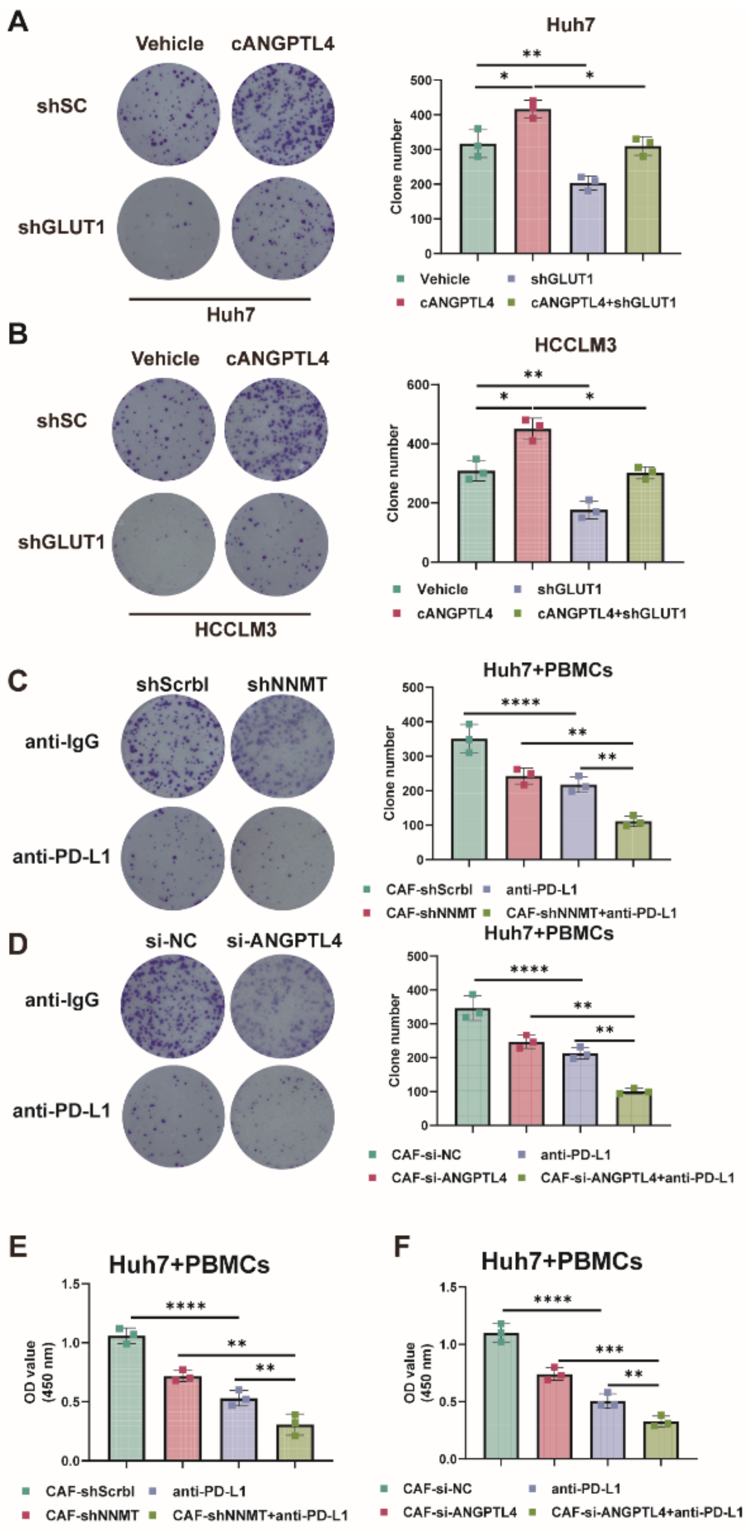

Supplementary Figure 9

The NNMT–ANGPTL4 axis promotes HCC cell proliferation and increases immune evasion.

A–B Representative images of colony formation assays for Huh7 **(A)** and HCCLM3 **(B)** cells following shGLUT1 and/or cANGPTL4 treatment.**C–D** Representative image

from the colony formation assays evaluating the proliferation of Huh7 cells following treatment with supernatant from NNMT-knockdown CAFs and(or) an anti-PD-L1 antibody with PBMCs. **E** CCK-8 assays evaluating the proliferation of Huh7 cells following treatment with supernatant with NNMT-knockdown CAFs and/or an anti-PD-L1 antibody. **F** CCK-8 assays evaluating the proliferation of Huh7 cells following treatment with supernatant from ANGPTL4-knockdown CAFs and/or an anti-PD-L1 antibody. (n=3; data are presented as the mean ± SD; $^*P < 0.05$, $^{**}P < 0.01$, $^{***}P<0.001$.)

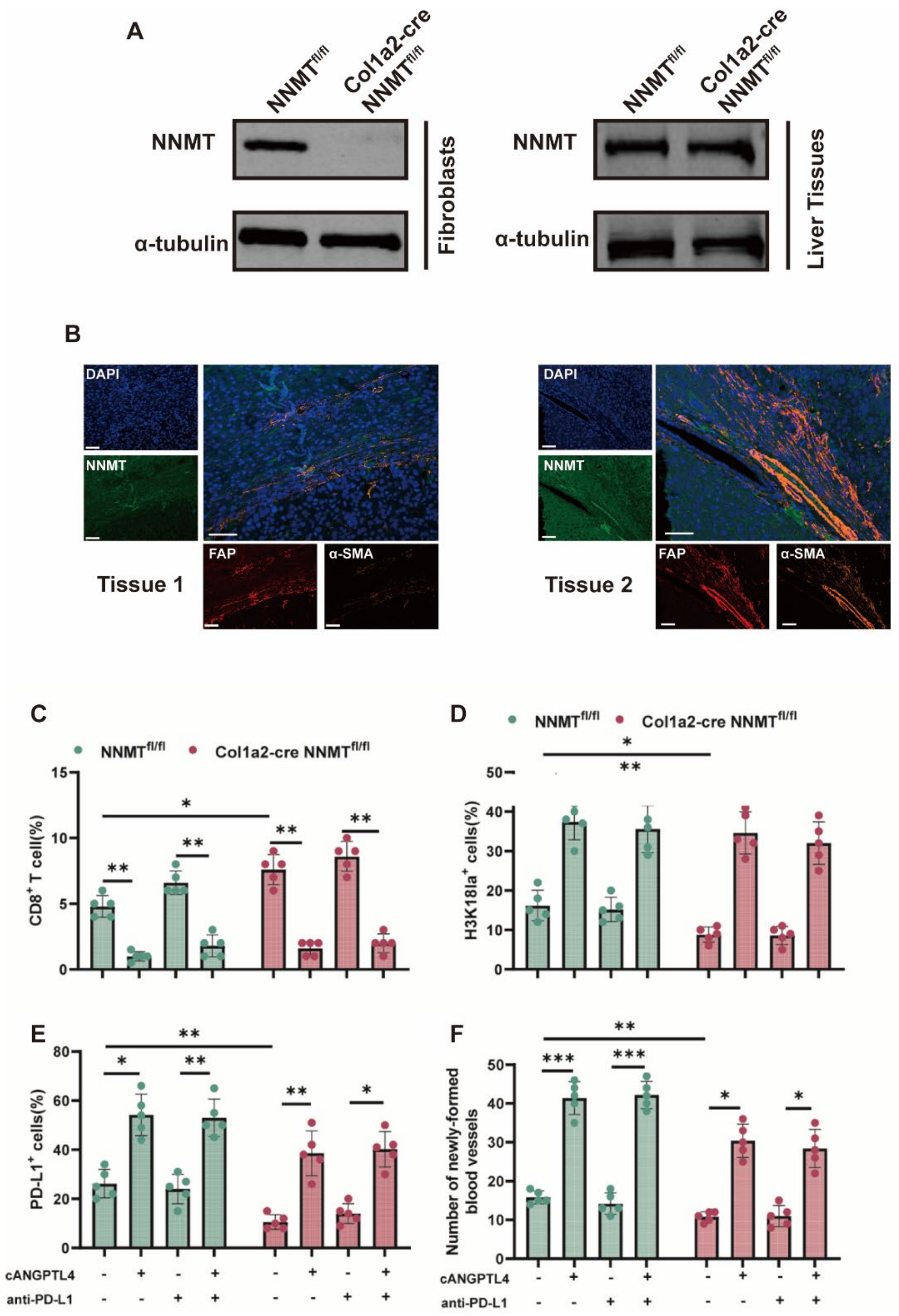

Supplementary Figure 10

The NNMT-ANGPTL4 axis promotes HCC progression in vivo.

A NNMT expression in liver tissues and fibroblasts from $NNMT^{fl/fl}$ mice and $Col1a2^{Cre}NNMT^{fl/fl}$ mice. **B** Localization of NNMT expression in liver cancer tissues from mice with fibroblast-specific knockout of NNMT. **C-F** Immunohistochemical staining was used to analyze CD8 **(C)**, PD-L1 **(D)**, H3K18la **(E)** and angiogenesis **(F)**. (n=5; data are presented as the mean ± SD; $^{*}P < 0.05$, $^{**}P < 0.01$, $^{***}P<0.001$.)

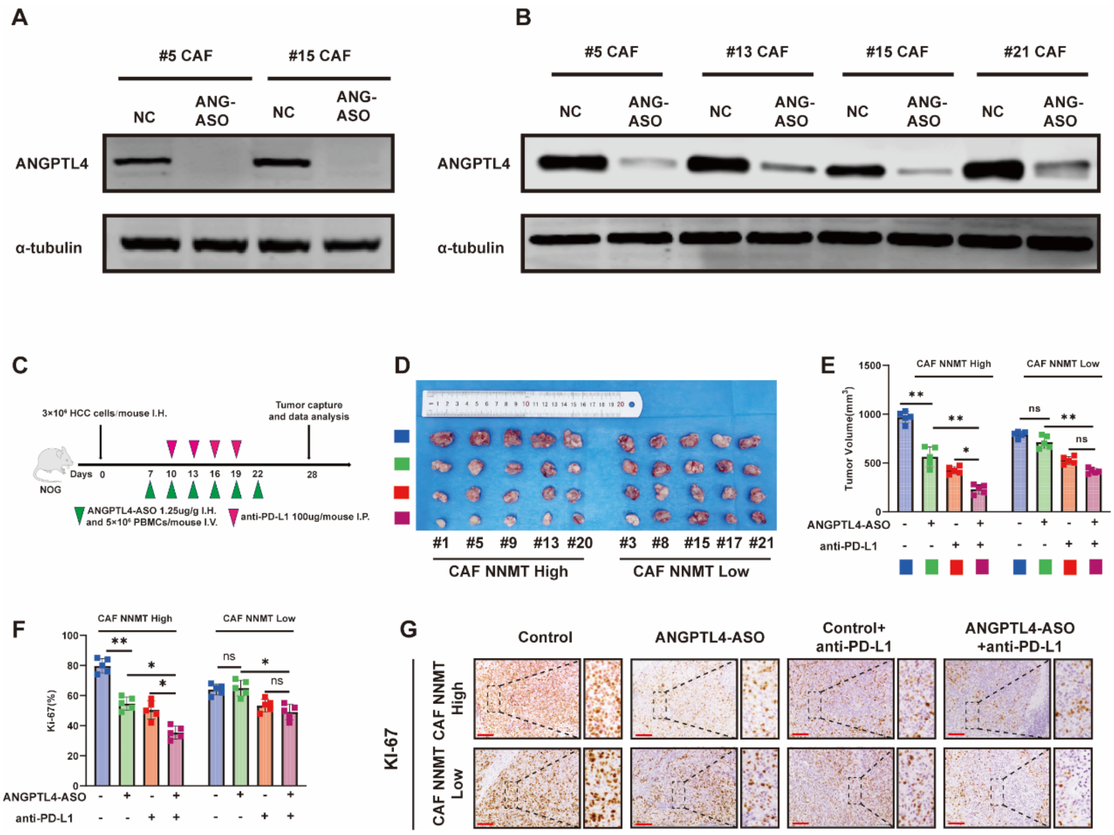

Supplementary Figure 11

Targeting ANGPTL4 expression suppresses HCC tumor progression.

A Intracellular ANGPTL4 expression in fibroblasts following transfection with the ANGPTL4-ASO sequence; **B** Effect of ANGPTL4-ASO on ANGPTL4 expression in cancer-associated fibroblasts (CAFs) within tumor tissues. **C** NOG mice were subcutaneously transplanted with the indicated PDX tissues and treated intravenously with ANGPTL4-ASO alone, an or with anti-PD-L1 antibody, or both starting on day 7 after transplantation. (n=5). **D** Tumor volumes of subcutaneous xenografts of PDX tissues. E Representative images of subcutaneous xenografts of PDX tissues. **F–G** KI-67 positivity rate (F) and representative images (G) of IHC staining for Ki-67 in subcutaneous xenograft models of PDX tissues. (Data are presented as the mean ± SD,; *P < 0.05, **P < 0.01.)

Supplementary table 1 Primer information

Gene Name	Sequence (5′-3′)
NNMT	F: AGCTGGAGAAGTGGCTGAAG
	R: TGGACCCTTGACTCTGTTCC
ANGPTL4	F: GTCCACCGACCTCCCGTTA
	R: CCTCATGGTCTAGGTGCTTGT
PD-L1	F: TGGCATTTGCTGAACGCATTT
	R:TGCAGCCAGGTCTAATTGTTTT
beta-ACTIN	F: CCAAGGCCAACCGCGAGAAGATGAC
	R: AGGGTACATGGTGGTGCCGCCAG
ANGPTL4-promoter-ChIP-1	F: CAAGACTCCTCCGCCCACTC
	R: CGGATCACAGTCGTGTGAGGA
ANGPTL4-promoter-ChIP-2	F: TAGGGGAATGGGTAGGGAAG
	R: AGTTCTCAGGCAGGTGGAGA
ANGPTL4-promoter-ChIP-3	F: ACCGACCTCCCGTTAGCCCC
	R: CGGAGAACCAGCCCTGGGGA
ANGPTL4-promoter-ChIP-1	F: TGTACTTACCTTCGAGTCTCT
	R: CTGAGGCTTGCTATTAACCA
ANGPTL4-promoter-ChIP-2	F: AGTCCTCAAGGCTCTTCA
	R: TTAGTTATGGTGGTCAGGAA
ANGPTL4-promoter-ChIP-3	F: GAAGGAAGGATGGTACTGATA
	R: GGTCTTGGAGGTCAACATT

ANGPTL4-ASO	+C*+T*+C*+A*+T*+G*+T*+T*+A*+G*+G*+T*+A*+G*+G*+T*+T
NC-ASO	+C*+G*+T*+T*+T*+A*+G*+G*+C*+T*+A*+T*+G*+T*+A*+C*+ T*+T
Angptl4-ASO	+C*+T*+C*A*T*G*T*T*A*G*G*T*A*G*+G*+T*+T
Control-ASO	+C*+G*+T*T*T*A*G*G*C*T*A*T*G*T*A*+C*+T*+T

Supplementary table 2 Antibody information

Antibody	Host	Product number	Company	Dilution
NNMT	Rabbit	OAAN01939	Aviva System Biology	1:1000(WB)
				1:200(IHC)
				1:50(IP)
NNMT	Mouse	ab119758	Abcam	1:150(IF)
ANGPTL4	Rabbit	18374-1-AP	Proteintech	1:1000(WB)
				1:100(IP)
				1:100(IF)
α-SMA	Rabbit	#19245	Cell Signaling Technology	1:200(IF)
α-SMA	Mouse	67735-1-Ig	Proteintech	1:200(IF)
PD-L1	Rabbit	ab205921	abcam	1:1000(WB)
				1:200(IF)
				1:200(IHC)
GLUT1	Rabbit	21829-1-AP	Proteintech	1:1000(WB)
				1:50(IP)
EZH2	Rabbit	#5246	Cell Signaling Technology	1:1000(WB)
				1:100(CHIP)
				1:100(IP)
				1:100(IF)
MCT1	Rabbit	20139-1-AP	Proteintech	1:1000(WB)
				1:50(IP)

MCT4	Rabbit	22787-1-AP	Proteintech	1:1000(WB)
				1:50(IP)
H3	Rabbit	#4499	Cell Signaling Technology	1:2000(WB)
H3K4me3	Rabbit	# 9751	Cell Signaling Technology	1:1000(WB)
H3K9me3	Rabbit	#13969	Cell Signaling Technology	1:1000(WB)
H3K27me3	Rabbit	#9733	Cell Signaling Technology	1:1000(WB)
				1:100(CHIP)
H3K36me3	Rabbit	#4909	Cell Signaling Technology	1:1000(WB)
H3K18la	Rabbit	PTM-1406RM	PTM BIOLABS	1:800(WB)
				1:50(IHC)
H3K18la	Rabbit	PTM-1427RM	PTM BIOLABS	1:50(CHIP)
FLAG	Rabbit	#14973	Cell Signaling Technology	1:1000(WB)
				1:50(IP)
CD31	Rabbit	ab182981	abcam	1:200(IHC)
Ki-67	Mouse	#9449	Cell Signaling Technology	1:500(IHC)
GAPDH	Mouse	60004	Proteintech	1:10000(WB)
α-Tubulin	Mouse	66031	Proteintech	1:10000(WB)
